# Treatment of Advanced Gastro-Entero-Pancreatic Neuro-Endocrine Tumors: A Systematic Review and Network Meta-Analysis of Phase III Randomized Controlled Trials

**DOI:** 10.3390/cancers13020358

**Published:** 2021-01-19

**Authors:** Claudio Ricci, Giuseppe Lamberti, Carlo Ingaldi, Cristina Mosconi, Nico Pagano, Laura Alberici, Valentina Ambrosini, Lisa Manuzzi, Fabio Monari, Deborah Malvi, Francesca Rosini, Francesco Minni, Davide Campana, Riccardo Casadei

**Affiliations:** 1 Division of Pancreatic Surgery, Azienda Ospedaliero-Universitaria Di Bologna, via Albertoni 15, 40138 Bologna, Italy; carlo.ingaldi@gmail.com (C.I.); lau.alberici@gmail.com (L.A.); francesco.minni@unibo.it (F.M.); riccardo.casadei@unibo.it (R.C.); 2 Department of Internal Medicine and Surgery (DIMEC), Alma Mater Studiorum, University of Bologna, 40126 Bologna, Italy; 3 Center of Excellence for the Diagnosis and Cure of Neuroendocrine Neoplasms Certified by the European Neuroendocrine Tumor Society (ENETS), Azienda Ospedaliero-Universitaria Di Bologna, via Albertoni 15, 40138 Bologna, Italy; lamberti.giu88@gmail.com (G.L.); cristina.mosconi@aosp.bo.it (C.M.); nico.pagano@aosp.bo.it (N.P.); valentina.ambrosini@unibo.it (V.A.); lisa.manuzzi@gmail.com (L.M.); fabio.monari@aosp.bo.it (F.M.); deborah.malvi@aosp.bo.it (D.M.); francesca.rosini@aosp.bo.it (F.R.); davide.campana@unibo.it (D.C.); 4 Department of Specialistic, Diagnostic and Experimental Medicine (DIMES), Alma Mater Studiorum, University of Bologna, 40126 Bologna, Italy; 5 Division of Oncology, Azienda Ospedaliero-Universitaria Di Bologna, via Albertoni 15, 40138 Bologna, Italy; 6 Division of Radiology, Azienda Ospedaliero-Universitaria Di Bologna, via Albertoni 15, 40138 Bologna, Italy; 7 Division of Gastroenterology, Azienda Ospedaliero-Universitaria Di Bologna, via Albertoni 15, 40138 Bologna, Italy; 8 Division of Nuclear Medicine, Azienda Ospedaliero-Universitaria Di Bologna, via Albertoni 15, 40138 Bologna, Italy; 9 Division of Radiometabolic Therapy, Azienda Ospedaliero-Universitaria Di Bologna, via Albertoni 15, 40138 Bologna, Italy; 10 Division of Pathologhy, Azienda Ospedaliero-Universitaria Di Bologna, via Albertoni 15, 40138 Bologna, Italy

**Keywords:** neuroendocrine neoplasms, network metanalysis, carcinoid

## Abstract

**Simple Summary:**

The most effective and safest approach for the treatment of advanced gastro-entero-pancreatic neuroendocrine neoplasms (GEP–NENs) remains unknown. A systematic review was done to clarify this point. A network meta-analysis was used to overcome the multiarm problem. Our study confirmed that somatostatin analogs (SSAs) alone remain the best choice for well-differentiated GEP–NENs. ^177^Lu-Dotatate plus SSA is a valid alternative for midgut NENs since it has been shown to be slightly more efficacious but yielding a higher risk for toxicity than SSAs.

**Abstract:**

Several new therapies have been approved to treat advanced gastro-entero-pancreatic neuroendocrine neoplasms (GEP–NENs) in the last twenty years. In this systematic review and meta-analysis, we searched MEDLINE, ISI Web of Science, and Scopus phase III randomized controlled trials (RCTs) comparing two or more therapies for unresectable GEP–NENs. Network metanalysis was used to overcome the multiarm problem. For each arm, we described the surface under the cumulative ranking (SUCRA) curves. The primary endpoints were progression-free survival and grade 3–4 of toxicity. We included nine studies involving a total of 2362 patients and 5 intervention arms: SSA alone, two IFN-α plus SSA, two Everolimus alone, one Everolimus plus SSA, one Sunitinib alone, one ^177^Lu-Dotatate plus SSA, and one Bevacizumab plus SSA. ^177^Lu-Dotatate plus SSA had the highest probability (99.6%) of being associated with the longest PFS. This approach was followed by Sunitinib use (64.5%), IFN-α plus SSA one (53.0%), SSA alone (46.6%), Bevacizumab plus SSA one (45.0%), and Everolimus ± SSA one (33.6%). The placebo administration had the lowest probability of being associated with the longest PFS (7.6%). Placebo or Bevacizumab use had the highest probability of being the safest (73.7% and 76.7%), followed by SSA alone (65.0%), IFN-α plus SSA (52.4%), ^177^Lu-Dotatate plus SSA (49.4%), and Sunitinib alone (28.8%). The Everolimus-based approach had the lowest probability of being the safest (3.9%). The best approaches were SSA alone or combined with ^177^Lu-Dotatate.

## 1. Introduction

Several new therapies have been approved to treat well-differentiated advanced gastro-entero-pancreatic neuroendocrine neoplasms (GEP–NENs) in the last twenty years [1]. European Neuro-Endocrine Tumor Society (ENETS) and North American Neuro-Endocrine Tumor Society (NANETS) guidelines [2,3,4] recommend somatostatin analogs (SSAs) alone as first-line therapy. Interferon-α, tyrosine kinase inhibitors (Everolimus and Sunitinib), or peptide receptor radionuclide therapies (PRRT) are considered only in patients with progressive diseases as a second or further line of treatment. A recent network meta-analysis (NMA), including both phase II and III randomized clinical trials (RCTs), has tried to clarify the hierarchy of these approaches [5]. The authors suggested that combination therapies might be more effective than single-agent strategies, but the metanalysis failed in identifying the best approach (safest and more efficacious), the result being only partially conclusive. The reasons for this can be related to some methodological choices: (I) the inclusion of both phase II and III RCTs; (II) the lack of metaregression analysis to study the inconsistency or heterogeneity of the network; (III) the excessive scattering into several arms in building the network. Consequently, the last point, the results of Everolimus, were scattered in two arms, reducing the network’s power. Since the question about which is the most effective and safest approach for the treatment of advanced GEP–NENs remain unanswered, we performed an NMA, including only phase III RCTs.

## 2. Material and Methods

According to the Cochrane recommendations, a systematic review was done, and the paper was structured point-by-point following the PRISMA checklist (Preferred Reporting Items for Systematic Reviews and Meta-Analyses) [6].

### 2.1. Eligibility Criteria

Population Intervention Control Outcomes Studies (PICOS) methodology was used to define the eligibility criteria [7]:

(a) “Population” was represented by the patients having nonresectable GEP–NENs;
(b) “Intervention” arms were any nonsurgical therapy;
(c) the “Control” group was the placebo arm;
(d) all studies reporting at least PFS and grade 3–4 toxicity;
(e) all phase III RCTs included at least two arms.

NMA was used to overcome the multiarm problem. The arms, including combined therapies, were clustered together with monotherapy if the experimental approach was the same.

### 2.2. Information Source, Search, Study Selection, and Data Collection Process

PubMed, ISI Web of Science, and Scopus were used for the research. The last search was carried out on 1 October 2020. Details about the information sources, search, study selection, and data collection process are described in the Supplementary Materials.

### 2.3. Data Items

The following data were extracted to describe the characteristics of each study: first author, year of publication, acronym, affiliation/country, NEN population, previous treatment with SSAs, chemotherapy (CHT) or other therapy, previous resection of the primary tumor, study design, the sample size of each arm, and the outcomes of interest reported. As the primary endpoints, we evaluated (a) PFS as a measure of efficacy; (b) grade 3 and 4 of toxicity as a measure of safety [8]. For the PFS calculation, we measured the incidence density rate (number of events for “at-risk patients” per unit of time) to overcome different follow-up duration problems. This measure can be assimilated to the hazard rate (HR) for the patients exposed. The rate ratio (RR) obtained from the ratio of two incidence density rates can be assimilated to the HR only for the exponential model (constant hazard functions) and in the absence of large differences in the average follow-up duration between the groups [9]. Dedicated software was used (GetData Graphical Digitizer^@^ version 2.26) to extract the crude number of events and the period of observation from the Kaplan–Meier curves. The secondary efficacy-related endpoints were (a) rate of objective radiological response (ORR), defined according to RECIST 1.0 or 1.1 as the sum of partial and complete response (PR + CR) [9,10]; (b) rate of progressive disease (PD), according to RECIST 1.0 or 1.1 [10,11]; (c) overall survival. As secondary endpoints of safety, we evaluated (a) adverse events (AEs) and serious adverse events (SAEs) [8]; (b) “on-treatment” deaths (OTDs) and deaths drug-related (DDR), defined as deaths from any cause and deaths related to drug administration, respectively; (c) drug discontinuation due to AEs (DDAEs).

### 2.4. Geometries of the Network and Risk of Bias within the Individual Study

Network geometry was plotted using nodes and edges for arms and direct comparisons, respectively. The network geometries were visually explored to evaluate the presence of one or more common nodes. The risk of bias within the individual studies was evaluated using a revised tool for assessing the risk of bias in randomized trials (Rob2) [12].

### 2.5. Summary Measurements and Methods of the Analysis

We described the surface under the cumulative ranking (SUCRA) curves and mean ranks for each arm. They measure the probability, without uncertainty, expressed in percentages, that each treatment would be the best, based on the outcome analyzed [13,14,15,16]. The SUCRA values were also reported as an efficacy-safety plot (grade 3 and 4 of toxicity and PFS, respectively) [16]. Additional details of the statistical method are described in the Supplementary Materials.

### 2.6. Inconsistency, Risk of Bias across the Studies, and Meta-Regression Analysis

Consistency is the statistical manifestation of the transitivity through each node. The reliability of the network was evaluated, and inconsistency (*p*-value < 0.05) was tested with both the general approach (chi-square test) and the loop approach (ratio of odds ratio) [17]. Heterogeneity was evaluated and reported as tau (τ) [18]. τ-values > 0.6 were considered relevant. When inconsistency or relevant heterogeneity was observed, a multivariate meta-regression analysis was carried out to identify the factors having a non-negligible effect (*p*-value < 0.05). Publication/reporting bias was reported using an adjusted funnel plot, which was tested using the Egger and Begg tests [19] (*p*-value < 0.05).

## 3. Results

### 3.1. Studies Selected

The systematic search of the literature, following the PRISMA statement, is reported in Figure S1. Nine studies [20,21,22,23,24,25,26,27,28,29,30,31] were eligible for quality assessment and quantitative synthesis. Upon reviewing the data extraction, there was 100% agreement between the two reviewers.

### 3.2. Study Characteristics and Risk of Bias within Studies

The characteristics of the studies selected are summarized in Table 1 (population and design), Table 2 (clusters, endpoints, and risk of bias evaluation), Table S1 (the rationale for using various treatments), and Table 2 (covariates as a potential source of bias and heterogeneity). The quality of the included studies is reported in Figure S2. In order to perform the NMA, the patients were clustered into the following arms: 531 (22.5%) in the placebo arm, 520 (22%) in the SSA-alone arm, 628 (26.6%) in the Everolimus ± SSA arm, 86 (3.6%) in Sunitinib-alone arm, 116 (4.9%) in ^177^Lu-Dotatate plus SSA arm, 267 (11.3%) in IFN-α plus SSA arm, and 214 (9.1%) in Bevacizumab plus SSA arm (Table 2).

### 3.3. Network Structures and Geometries

**Figure 1:**
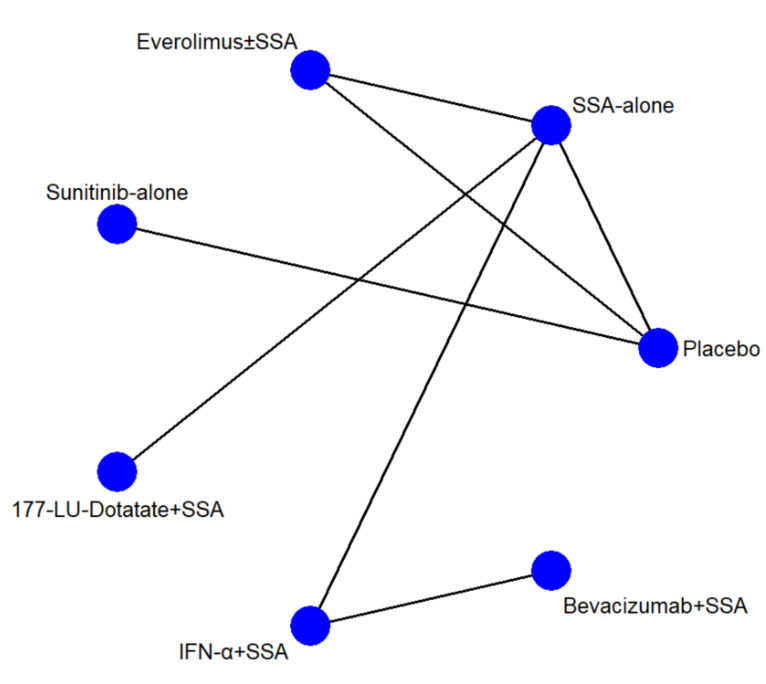
Network geometry. The blue circles represent the treatment arm, while the edges indicate the direct comparison available in the literature. SSA = somatostatin analog; ^177^-Lu = Lutezio; IFN-α = interferon alfa.

The network geometries (Figure 1) were the same for all endpoints except for the AE network, in which the IFN-α and Bevacizumab arms were lacking. There were seven arms and seven types of direct comparisons. We found only one triangular loop (SSA vs. Everolimus ± SSA vs. Placebo). On primary endpoints, the most informative direct evidence was SSA vs. IFN-α plus SSA, with a contribution of 21.6% and 20.5% to the entire network for PFS and toxicity grade 3 and 4, respectively (Figure S3A,B). The remaining contribution plots are reported in Figure S3C–J.

### 3.4. Synthesis of Results

The SUCRA and the mean rank values for each scenario are shown in Table 3, while the relative ranking probabilities are displayed in Table S3. The “head-to-head” comparisons for all endpoints are plotted in Figure S4A–J, respectively.

#### 3.4.1. Efficacy (PFS)

The pooled event rates were 1718 out of 2362 (72.7%). The events per 100 patients–one year were 48, 26, 31, 51, 10, 26, and 27 in the placebo, SSA-alone, Everolimus ± SSA, Sunitinib-alone, ^177^Lu-Dotatate plus SSA, IFN-α plus SSA, and Bevacizumab plus SSA arms, respectively. The NMA suggests that a treatment based on ^177^Lu-Dotatate plus SSA has the highest probability (99.6%) of being associated with the longest PFS, and, on average, this approach is always the best possible. This approach is followed by the use of Sunitinib (SUCRA = 64.5), IFN-α plus SSA (SUCRA = 53.0;), SSA-alone (SUCRA = 46.6), Bevacizumab plus SSA one (SUCRA = 45.0), and Everolimus ± SSA (SUCRA = 33.6). The placebo administration had the lowest probability of being associated with the longest PFS (SUCRA value = 7.6).

#### 3.4.2. Safety (Toxicity Grade 3 and 4)

The pooled event rates were 907 for the 2362 patients exposed (38.4%). The event rate was 23.7%, 25.0%, 41.7%, 65.1%, 39.7%, 59.9%, and 59.3% in the placebo, SSA-alone, Everolimus ± SSA, Sunitinib-alone, ^177^Lu-Dotatate plus SSA, IFN-α plus SSA, and Bevacizumab plus SSA arms, respectively. The NMA suggests that a treatment based on placebo or Bevacizumab use has the highest probability of being the safest (SUCRA values of 73.7 and 76.7, respectively). These approaches are followed by SSA-alone (SUCRA = 65.0), IFN-α plus SSA (SUCRA = 52.4), ^177^Lu-Dotatate plus SSA (SUCRA = 49.4), and Sunitinib-alone (SUCRA = 28.8). The Everolimus-based approach has the lowest probability of being the safest (SUCRA value = 3.9).

#### 3.4.3. Safety/Efficacy Ratio

**Figure 2:**
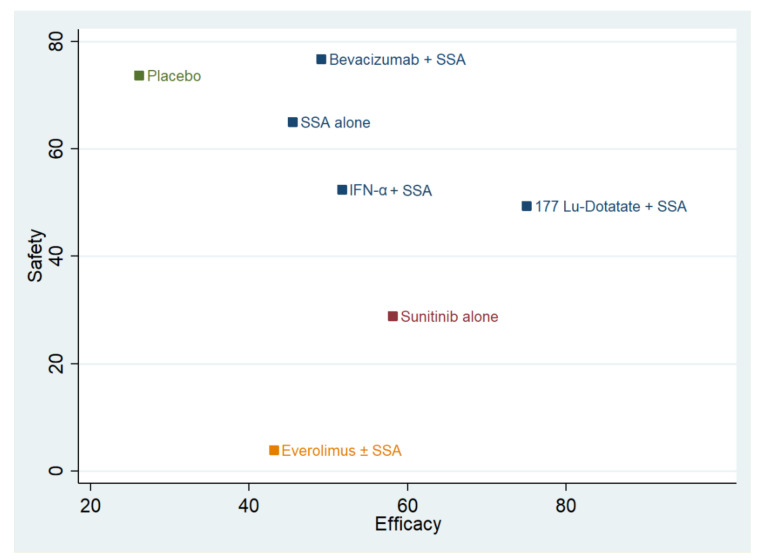
Cluster rank combining surface under the cumulative ranking curve (SUCRA) values. The *y*-axis reports the SUCRA values as a percentage of “safety” (toxicity 3 and 4). The *x*-axis reports the efficacy (progression-free survival). Different colors identify the different clusters. SSA = somatostatin analog^; 177^-Lu-Dotatate = Lutezio with Dotatate; IFN-α = interferon alfa.

The combination plot is reported in Figure 2. The best approaches are SSA-alone and SSA in combination with Bevacizumab or IFN-α or ^177^Lu-Dotatate. The second choice is Sunitinib use, followed by Everolimus and placebo. The cophenetic correlation coefficient was 0.93. The maximum value of clustering gain was 447.31, and the optimal number of clusters was 4.

#### 3.4.4. Secondary Endpoints

The results of all secondary endpoints are exhaustively reported in the Supplementary Materials. The treatment with the highest probability of improving OS is Sunitinib, followed by ^177^Lu-Dotatate plus SSA, with a SUCRA value of 93.6 and 87.7, respectively. The approach with the highest probability of obtaining an ORR is Bevacizumab plus SSA (SUCRA = 88.3). The therapy with the highest probability of preventing the disease’s radiological progression is ^177^Lu-Dotatate plus SSA (SUCRA = 90.6). The treatment with the highest probability of avoiding any AEs is SSA-alone (SUCRA = 93.6). Considering SAEs, the worst approach, according to the model, is Bevacizumab plus SSA therapy (SUCRA = 0). The approach with the lowest probability of being related to OTD is Sunitinib (SUCRA = 87.3). DDR incidence could be higher when the therapy is based on Everolimus (SUCRA = 42.0), IFN-α (SUCRA = 37.7), or Bevacizumab (SUCRA = 32.4). The approach with the highest probability of avoiding “drug-discontinuation” is ^177^Lu-Dotatate (SUCRA = 86.7).

#### 3.4.5. Inconsistency, Heterogeneity, and Publication Bias

Inconsistency and heterogeneity for all outcomes are shown in Table 4. A significant inconsistency was found for PFS (chi-square = 4.86; *p* = 0.027) due to the lack of transitivity across the nodes that were attributable to the closed-loop, including the direct comparison between placebo, SSA-alone, and Everolimus (RoR = 1.78; *p* = 0.016). This loop and the relative direct and indirect information are plotted in Figure 2. No relevant heterogeneity was found for the other endpoints. The meta-regression analysis (Table S4) explains the inconsistency: SSA-alone efficacy was overestimated when treatment-naïve patient rates were high (SMD = 0.55; *p* = 0.028).

### 3.5. Primary Endpoints

On the contrary, underestimating SSA-alone efficacy could be attributable to poor balancing between the two arms of patients with extrahepatic metastatic disease (SMD = −0.74: *p* = 0.002). According to the metaregression analysis, the adjusted SUCRA and rank values reported in Table 4 show that ^177^Lu-Dotatate has the highest chance of being associated with the longest PFS, followed by IFN-α plus SSA and Bevacizumab plus SSA. No “small-study” effect was found using the Begg and Egger tests, as showed by the funnel plots (Figure S5A–J).

## 4. Discussion

Our study demonstrates that SSA, alone or in combination with ^177^Lu-Dotatate, can be considered the best approach in terms of efficacy measured by PFS for unresectable GEP–NENs. We included 9 phase III RCTs and 2362 patients. In contrast to the paper of Kaderli et al. [5], we used stringent inclusion criteria. Moreover, we used a different way to build the network: all Everolimus-based arms, with or without SSAs, were clustered together to avoid an excessively scattered network. The analysis suggests some interesting considerations. First, the best-suggested approach is ^177^Lu-Dotatate plus SSA because the probability that this therapeutic choice is related to the longest event-free lifespan is the highest and near to the ideal approach. An excellent second choice seems to be Sunitinib’s use alone, at least in Pan-NENs, in which this drug is approved. SSA, alone or combined with Bevacizumab and IFN-α, seems to have equal chances of prolonging PFS, resulting in being the third preferred choice for Pan-NENs and the second for gastrointestinal NENs.

**Figure 3:**
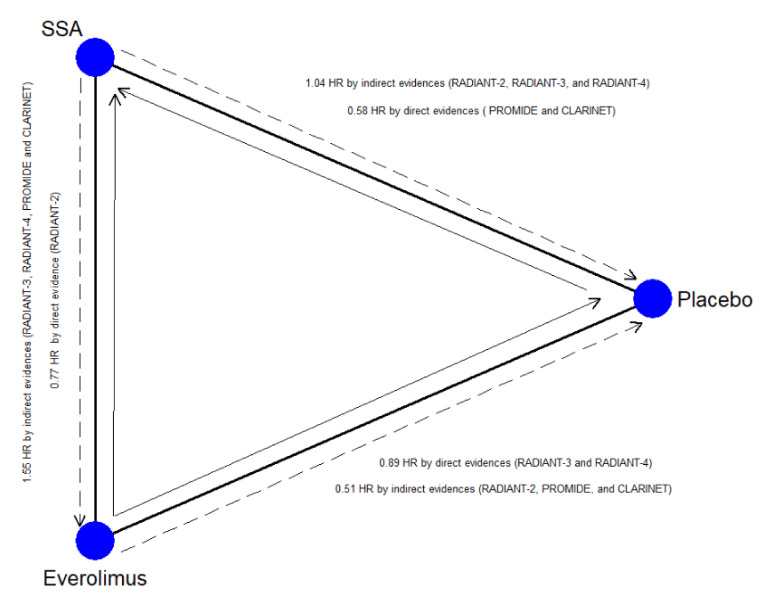
Closed-loop within the network. The blue circles represent the treatment arm while the edges indicate the direct comparison available in the literature SSA = Somatostatin Analog; the dashed arrow represents the indirect estimates; the full arrow the was the direct evidence; the tip of the arrow indicates the therapy more efficacious as Progression-Free Survival.

On the contrary, Everolimus ± SSA is a worse choice two times out of three. Nevertheless, this therapy remains superior to placebo. The validity of these results is weakened by the network’s inconsistency, namely, by incoherence among direct and indirect estimates [32]. The main source of inconsistency is located within the closed-loop Placebo vs. SSA alone vs. Everolimus ± SSA (Figure 3). The reason is well known among oncologists and physician experts in the treatment of GEP–NENs [33]. The rate of treatment-naïve patients (with stable disease at the time of enrollment and without previous treatment), primary tumor site, and the rate of patients with extrahepatic disease were some factors that could have been imbalanced between the arms of the RADIANT-3 and RADIANT-4 [25,26,29], CLARINET [28], and PROMID [21,22] studies. For example, PFS at 24 months in the placebo arm of the CLARINET trial [28] (high rate of naïve patients, all GEP–NENs included) was 33%, while in the RADIANT-3 trial (low rate of naïve patients, only Pan-NENs), it was 0%. We statistically studied this phenomenon and recognized the reason for the inconsistency. PFS of the placebo arm was statistically higher when the rate of treatment-naïve patients increased. The placebo arm is a crucial common node for the indirect comparison of SSA-alone vs. Everolimus ± SSA. Thus, the indirect comparisons overestimated the effect of SSA and underestimated the effect of Everolimus. The network’s weakness was corrected, weighing the effect of all potential confounding factors with meta-regression analysis. Considering the adjusted SUCRA values, ^177^Lu-Dotatate plus SSA still remains the best choice.

It should be noted that absolute efficacy was reduced (rank 2.6) for an ideal therapy. Even though ^177^Lu-Dotatate plus SSA was tested in a phase III RCT for midgut NENs only, it has been registered by the FDA [34] and the EMEA [35] to treat well-differentiated Pan-NENs. The remaining approaches were far from ideal therapy (rank around 4).

The analysis of the rate of grade 3 and 4 toxicity permits some further considerations. Excluding the placebo arm, the best approach should be chosen among SSA alone or combined with Bevacizumab, IFN-α, or ^177^Lu-Dotatate. Thus, combining the safety and efficacy data, in contrast to the papers of Kaderly et al. [5], we did not observe a generic superiority of combination therapies compared to monotherapy. ^177^Lu-Dotatate plus SSA, even with a slight increase in toxicity, was the choice with the best combination of efficacy/safety. The Sunitinib and Everolimus ± SSA arms were penalized by the low probability of being the safest, rather than their efficacy. Moreover, in a recent paper by Mujica-Mota et al. [36], Everolimus was considered less cost-effective than Sunitinib or ^177^Lu-Dotatate plus SSA.

The analysis of secondary endpoints suggested that each approach could have some advantages. Bevacizumab plus SSA, Sunitinib, and ^177^Lu-Dotatate plus SSA have the best chance of obtaining an ORR, suggesting that these approaches might be used to reduce the symptoms related to tumor volume. However, ^177^Lu-Dotatate and Bevacizumab are associated with a higher chance of achieving radiological disease stability than Sunitinib. ^177^Lu-Dotatate plus SSA and Sunitinib-alone seem to be related to the highest possibility of longer survival. Nevertheless, any speculation regarding overall survival should be interpreted with caution due to the high crossover rate between arms. Furthermore, ^177^Lu-Dotatate has the lowest probability of discontinuation for SAEs, even if SSA-alone is the best choice for AEs and the second-best choice for SAEs, confirming the high tolerability of this approach. Finally, it should be noted that approaches based on Everolimus or IFN-α were never among the three first choices for all secondary endpoints.

This analysis has some limitations. First, the populations included were heterogeneous for several important factors. Remarkably, the rate of treatment-naïve patients was very high in some arms, such as placebo or SSA-alone, compared to others. The last bias was the main reason for the inconsistency for the primary endpoint. Nonetheless, with the proper use of the metaregression analysis, this effect was recognized and corrected, presenting the results in a credible clinical way. Third, some RCTs included only either gastrointestinal NENs (^177^Lu-Dotatate or Bevacizumab) or Pan-NENs (Sunitinib). Thus, the applicability of some clinical practice results could be limited: for example, Sunitinib is not approved for midgut NENs. Nevertheless, the same network inclusion was possible using the metaregression analysis, which demonstrated that the type of tumors did not influence each approach’s adjusted value for all endpoints. Moreover, some treatments are available in clinical practice for both gastrointestinal and Pan-NENs, such as ^177^Lu-Dotatate. Furthermore, all information obtained from the network could have scientific value, providing useful research data to design future trials. Fourth, the network analysis required some approximations: (I) the Everolimus cluster included both Everolimus and Everolimus plus SSA arms; (II) the SSA cluster included several approaches, such as thrice daily or several types of long-acting octreotide (Octreotide LAR 30 or 60 mg, Lanreotide 120 mg, depot. Octreotide 20 mg); (III) the placebo arm could have included, in some cases, the on-demand use of SSAs. Nonetheless, these approximations were necessary to avoid an excessive scattering of the network. Fifth, no recent studies that included a chemotherapy arm is available for the analysis in the network. Finally, the included trials used different tumor grade systems due to WHO classification changes over the past decades. Nonetheless, all studies included well-differentiated neuroendocrine tumors.

In conclusion, our study confirms that SSA-alone, as suggested by ENETS and NANETS guidelines, remains the best choice for well-differentiated GEP–NENs. ^177^Lu-Dotatate plus SSA is a valid alternative for midgut NENs, since it has been shown to be slightly more efficacious but yielding a higher risk for toxicity than SSA. Further studies, such as the NETTER-2 trial [37], could confirm the efficacy of PRRT as a first-line treatment in Pan-NENs as well. SSA combination therapy with Bevacizumab or IFN-α did not significantly increase the efficacy and worsened the safety in IFN-α. Sunitinib and Everolimus approaches remain the second choice because we did not observe a significant increase in efficacy to SSA use alone. The algorithm reported in Figure S6 summarizes the results of the meta-analysis.

## Figures and Tables

**Table 1:** Characteristics of the nine included studies.

First Author/Year	Acronyms	Affiliation/Country	Population ^$^	Pan-NET	Previous SSA Therapy	Previous CHT	Other Therapy	Previous Surgery of Primary Tumor	Design
Arnold et al., 2005 [20]	-	Multicenter	GEP-NET	Yes	Yes	Yes	No	^§^	Octreotide * vs. Octreotide * + IFN-α
Rinke et al., 2009 [21,22]	PROMIDE	Multicenter	Midgut NET	No	Yes ^#^	No	No	Yes	Placebo vs. Octreotide LAR 30 mg
Pavel et al., 2011 [23,24]	RADIANT-2	Multicenter	GEP-NET	Yes	Yes	Yes	Yes ^	^§^	Octreotide LAR 30 mg vs. Octretide LAR 30 mg + Everolimus 10 mg
Yao et al., 2011 [25,26]	RADIANT-3	Multicenter	Pan-NET	Yes	Yes	Yes	Yes ^^	^§^	Placebo vs. Everolimus 10 mg
Raymond et al., 2011 [27]	-	Multicenter	Pan-NET	Yes	Yes	Yes °°	Yes °°	Yes	Placebo vs. Sunitinib 37.5 mg
Caplin et al., 2014 [28]	CLARINET	Multicenter	GEP-NET	Yes	Yes ^£^	Yes ^£^	Yes ^£^	Yes ^£^	Placebo vs. Lanreotide 120 mg
Yao et al., 2016 [29]	RADIANT-4	Multicenter	Lung or Midgut NET	No	Yes	Yes	Yes	Yes	Placebo vs. Everolimus 10 mg
Strosberg et al., 2017 [30]	NETTER-1	Multicenter	Midgut NET	No	Yes **	Yes **	Yes **	Yes	^177^ Lu-Dotate + Octreotide LAR 30 mg vs. Octreotide LAR 60 mg
Yao et al., 2017 [31]	SWOG S0518	Multicenter	Midgut NET	No	Yes ^£^	Yes	Yes ^£^	Yes ^£^	depot Octreotide 20 mg + Bevacizumab vs. depot Octreotide 20 mg + IFN-α

Legend: ^$^ = all tumors were well-differentiated, even if classified with different tumor grading systems; * = 200 µg ocreotide thrice daily; ^#^ = pretreatment with somatostatin analogs for more than 4 weeks or previous treatment with interferon alfa, chemotherapy, or chemoembolization; ^ = only the patients with hepatic artery embolization or cryoablation were excluded; ° = best supportive care with SSA on-demand; ^^ = immunotherapy or radiotherapy within four weeks prior to starting this trial or the hepatic artery procedure called embolization within the last six months or cryoablation/radiofrequency ablation of hepatic metastasis within two months of enrollment; § = not reported; °° = chemotherapy, locoregional therapy (e.g., chemoembolization) or interferon at least four weeks before baseline assessment; ^£^ = patients excluded because they had received treatment with interferon, chemoembolization, or chemotherapy within 6 months before study entry, a radionuclide at any time, or a somatostatin analogue at any time; ** = any surgery, liver-directed transarterial therapy, or chemotherapy within 12 weeks before randomization or major surgery related to the neuroendocrine tumor within 3 months before study entry; £ = prior surgery, liver-directed therapy, and radiotherapy were allowed if completed more than 28 days before the start of study therapy, the patient had recovered from the procedure, and there were residual sites of measurable disease; prior depot octreotide was allowed, provided at least 21 days had elapsed since the last dose to the start of study therapy.

**Table 2:** Characteristics of the nine studies included.

First Author/Year	Clusters							Endpoints	Rob2
	Placebo (Arm A)	SSA-Based (Arm B)	Everolimus-Based (Arm C)	Sunitinib -Based (Arm D)	^177^Lu-Dotatate-Based (Arm E)	IFN-α-Based (Arm F)	BevacizumaB-Based (Arm G)		
Arnold et al., 2005 [20]	-	51	-	-	-	54	-	PFS, grade 3–4 toxicity, OS, ORR, PD, SAEs, OTD, DDR, DDAEs	Low risk
Rinke et al., 2009 [21,22]	43	42	-	-	-	-	-	PFS, grade 3–4 toxicity, OS, ORR, PD, SAEs, OTD, DDR, DDAEs	Some Comcerns
Pavel et al., 2011 [23,24]	-	213	216	-	-	-	-	PFS, grade 3–4 toxicity, OS, ORR, PD, SAEs, OTD, DDR, DDAEs	Some Comcerns
Yao et al., 2011 [25,26]	203	-	207	-	-	-	-	PFS, grade 3–4 toxicity, OS, ORR, PD, AEs, SAEs, OTD, DDR, DDAEs	Low risk
Raymond et al., 2011 [27]	85	-	-	86	-	-	-	PFS, grade 3–4 toxicity, OS, ORR, PD, AEs, SAEs, OTD, DDR, DDAEs	Low risk
Caplin et al., 2014 [28]	103	101	-	-	-	-	-	PFS, grade 3–4 toxicity, OS, PD, AEs, SAEs, OTD, DDR, DDAEs	Low risk
Yao et al., 2016 [29]	97	-	205	-	-	-	-	PFS, grade 3–4 toxicity, OS, ORR, PD, AEs, SAEs, OTD, DDR, DDAEs	Low risk
Strosberg et al., 2017 [30]	-	113	-	-	116	-	-	PFS, grade 3–4 toxicity, OS, ORR, PD, AEs, SAEs, DDR, DDAEs	Low Risk
Yao et al., 2017 [31]	-	-	-	-	-	213	214	PFS, grade 3–4 toxicity, OS, ORR, PD, SAEs, OTD, DDR, DDAEs	Low Risk
Total	531	520	628	86	116	267	214		

Legend: SSA = somatostatine analogues; ^177^Lu = Lutezio; IFN-Þ = interferon alfa; Rob2 = quality assessment using a revised Cochrane risk-of-bias tool for randomized trials; PFS = progression-free survival; OS = overall survival; ORR = objective radiological response; PD = progressive disease evaluated with imaging; AEs = adverse events (any type); SAEs = severe adverse events; OTDs = on treatment deaths; DDR = deaths drug-related; DDAEs = drug discontinuation due to adverse events.

**Table 3:** The surface under cumulative ranking area (SUCRA) values and mean rank for all outcomes. The SUCRA values express, in percentages, the safety or efficacy of each approach relative to an imaginary approach, which is always best without uncertainty.

Outcomes of Interest	Studies	SUCRA (%) and Rank (Mean) for Arm													
		Placebo		SSA Alone		Everolimus ± SSA		Sunitinib Alone		^177^Lu-Dotatate + SSA		IFN-α + SSA		Bevacizumab + SSA	
		SUCRA	Rank	SUCRA	Rank	SUCRA	Rank	SUCRA	Rank	SUCRA	Rank	SUCRA	Rank	SUCRA	Rank
Progression-Free Survival	9	7.6	6.5	46.6	4.2	33.6	5.0	64.5	3.1	99.6	1.0	53.0	3.8	45.0	4.3
Grade 3–4 toxicity °	9	73.7	2.4	65.0	3.1	3.9	6.8	28.8	5.3	49.4	4.0	52.4	3.9	76.7	2.4
Overall Survival	9	32.6	5.0	48.9	4.1	43.4	4.4	93.6	1.4	87.7	1.7	31.8	5.1	11.9	6.3
ORR (CR+PR) ^§^	8	6.9	5.8	20.0	5.8	32.0	5.1	74.2	2.5	68.6	2.8	59.0	3.5	88.3	1.7
PD ^§^	9	2.6	6.8	22.8	5.6	56.9	3.6	35.0	4.9	90.6	1.6	61.3	3.3	80.8	2.2
AEs	6	74.1	2.0	96.3	1.1	27.6	3.9	33.7	3.7	18.3	4.3	*	*	*	*
SAEs	9	60.6	3.4	76.4	2.4	31	5.1	96.5	1.2	65.6	3.1	19.9	5.8	0	7.0
On Treatment Deaths	8	61.2	2.9	56.8	3.2	15.9	5.2	87.3	1.6	34.9	4.3	43.8	3.8	*	*
Deaths Drug-related	9	64.3	3.1	58.5	3.5	42.0	4.5	58.5	3.5	56.7	3.6	37.7	4.7	32.4	5.1
Discontinuation for SAE	9	85.7	1.9	71	2.7	19.5	5.8	52.1	3.9	86.7	1.8	25	5.5	10	6.4

Legend: SUCRA = the surface under the cumulative ranking curve; SSA = somatostatin analog therapy; Lu = Lutetium; AE = adverse event, defined according to National Cancer Institute Common Terminology Criteria for Adverse Events; SAE = severe adverse event, defined according to National Cancer Institute Common Terminology Criteria for Adverse Events; Lu = Lutetium; ORR = objective radiological response; CR = complete response; PR = partial response; PD = progressive disease; § = evaluated according to Response Evaluation Criteria In Solid Tumors; * = not computable; ° = the datum was calculated as the number of events per patients; IFN-α = interferon alfa.

**Table 4:** Inconsistency, heterogeneity, and adjusted SUCRA and rank values.

Outcomes of Interest	Inconsistency					τ Value	Adjusted SUCRA and Rank Values													
	Global		Local				Placebo		SSA Alone		Everolimus ± SSA		Sunitinib Alone		^177^Lu-Dotatate + SSA		IFN-α + SSA		BevacizumaB + SSA	
	Chi-Square	*p*-Value	Loop	RoR	*p*-Value		SUCRA	Rank	SUCRA	Rank	SUCRA	Rank	SUCRA	Rank	SUCRA	Rank	SUCRA	Rank	SUCRA	Rank
PFS	4.86	0.027	ABC	1.78	0.016	0.2	26.1	5.4	45.5	4.3	43.1	4.4	58.1	3.5	75.0	2.6	51.7	3.9	49.1	4.1
Grade 3–4 toxicity	0.03	0.853	ABC	1.28	0.628	0.3	-	-	-	-	-	-	-	-	-	-	-	-	-	-
OS	0.52	0.470	ABC	1.27	0.443	0.1	-	-	-	-	-	-	-	-	-	-	-	-	-	-
ORR (CR + PR) ^§^	0.14	0.707	ABC	1.84	0.713	<0.1	-	-	-	-	-	-	-	-	-	-	-	-	-	-
PD ^§^	1.17	0.279	ABC	2.26	0.144	0.4	-	-	-	-	-	-	-	-	-	-	-	-	-	-
AE	1.75	0.185	ABC	1.30	0.185	<0.1	-	-	-	-	-	-	-	-	-	-	-	-	-	-
SAE	0.01	0.927	ABC	1.04	0.928	<0.1	-	-	-	-	-	-	-	-	-	-	-	-	-	-
On treatment deaths	0.02	0.883	ABC	1.18	0.877	<0.1	-	-	-	-	-	-	-	-	-	-	-	-	-	-
Deaths Drug-related	0.09	0.768	ABC	2.13	0.770	0.5	-	-	-	-	-	-	-	-	-	-	-	-	-	-
Discontinuation for SAE	0.30	0.585	ABC	1.72	0.600	<0.1	-	-	-	-	-	-	-	-	-	-	-	-	-	-

**Legend:** RoR = rate of odds ratio; SSA = somatostatin analogue therapy; Lu = Lutetium; IFN-α = interferon alfa; PFS = progression-free survival; OS = overall survival; SSA = somatostatin analogue therapy; AE = adverse event defined according to National Cancer Institute Common Terminology Criteria for Adverse Events; SAE = severe adverse event defined according to National Cancer Institute Common Terminology Criteria for Adverse Events; Lu = Lutetium; ORR = objective radiological response; CR = complete response; PR = partial response; PD = progressive disease; § = evaluated according to Response Evaluation Criteria In Solid Tumors;- = not computable.

## Data Availability

The data presented in this study are available on request from the corresponding author.

## Supplementary Materials

The following are available online at https://www.mdpi.com/2072-6694/13/2/358/s1; Supplementary Table S1. Covariates potentially source of bias and heterogeneity in included studies; Supplementary Table S2- The ranking of the therapies for all outcomes.; Supplementary Table S3- Meta-regression analysis for progression-free survival; Supplementary Figure S1. PRISMA Flow Diagram illustrating study selection; Supplementary Figure S2. Quality of included study; Supplementary Figure S3 (Panel A-J): Contribution plots of all outcomes. In the columns, all available direct comparisons (comparisons evaluated in at least one study) are reported while, in the rows, the following are reported: (1) the mixed comparisons (namely the estimates already available in the literature but implemented by the network) and (2) the indirect comparisons (namely the comparisons not available in the literature but generated by the network). The table should be read from left to right; each row contains the contribution of each direct comparison in the network (mixed and indirect) estimates and, thus, the cumulative sum of the contributions is 100 (in percentages). In the plots, the contribution of each direct comparison in building the entire network is also reportedPanel B: Grade 3-4 toxicity; Panel C: Overall survival; Panel D: Objective Radiological Response; Panel E: Progressive Disease; Panel F: Adverse Event; Panel G: Severe Adverse Event; Panel H: On treatment deaths; Panel I: Deaths Drug-related; Panel J: Discontinuation for SAE; Supplementary Figure S4 (Panel A-J): Forest plots of all outcomes. The results are reported as Odds ratios (ORs) and 95% confidence intervals (CIs). The blue line (line of null effect) is equal to 1. The solid black lines represent the CIs while the diamond summarises the ORs. For each pairwise comparison, the forest plot should be read as following: if the diamond with the entire CIs did not reach the blue line of null effect, there is a significant difference. If the entire CI is on the left of the null effect, the mortality rate is significantly higher in the “intervention arm” while, when the entire CI is on the right, the event is statistically more frequent in the “reference arm.” When the entire CI crosses the null effect line, the difference between the two procedures compared is not statistically significant. Besides, a red line reports the Predictive Interval (PrI), namely the interval within which the estimate of a future study is expected to be. Panel A: Progression-free survival; Panel B: Grade 3-4 toxicity; Panel C: Overall survival; Panel D: Objective Radiological Response; Panel E: Progressive Disease; Panel F: Adverse Event; Panel G: Severe Adverse Event; Panel H: On treatment deaths; Panel I: Deaths Drug-related; Panel J: Discontinuation for Severe Adverse Event; Supplementary Figure S5: Funnel plots of the network estimates of all outcomes. In the comparison-adjusted funnel plot, the horizontal axis shows the difference of each i-study estimate YiXY from the summary effect for the respective comparison (YiXY-μXY). In contrast, the vertical axis presents a measure of the dispersion of YiXY, namely the standard error of the effect size. The red line shows the null hypothesis. Each point represents a direct comparison: different colors correspond to different comparisons. The dashed black line represents the 95% confidence interval. The horizontal line represents the regression line; the sky blue regression line demonstrates no asymmetry; Arm 1 = Placebo; Arm 2 = octreotide analogs (SSA) alone; Arm 3 = Everolimus ± SSA; Arm 4 = Sunitinib; Arm 5 = 177Lu-Dotatate plus SSA; Arm 6 = Interferon alfa plus SSA; Arm 7 = Bevacizumab plus SSA. Panel A: Progression-free survival; Panel B: Grade 3-4 toxicity; Panel C: Overall survival; Panel D: Objective Radiological Response; Panel E: Progressive Disease; Panel F: Adverse Event; Panel G: Severe Adverse Event; Panel H: On treatment deaths; Panel I: Deaths Drug-related; Panel J: Discontinuation for Severe Adverse Event.
