# Supplementary material for: Treatment of Advanced Gastro-Entero-Pancreatic Neuro-Endocrine Tumors: A Systematic Review and Network Meta-Analysis of Phase III Randomized Controlled Trials
Source: Cancers (Basel). 2021 Jan 19;13(2):358. doi: 10.3390/cancers13020358 (PMC7835931; doi:10.3390/cancers13020358)
Supplement: Supplementary file 1 [file cancers-13-00358-s001.pdf]

cancers

MDPI

Supplementary Materials

Treatment of Advanced Gastro-Entero-Pancreatic Neuro-Endocrine Tumors: A Systematic Review and Network Meta-Analysis of Phase III Randomized Controlled Trials

Supplementary Methods

1. Information Sources and Search Strategy

A systematic literature search was conducted to identify studies. NMA was used to overcome the multi-arm problem. The arms, including combined therapies, were clustered together with monotherapy if the experimental approach was the same (e.g., the arms included the Everolimus approach were clustered together with Everolimus + SSA). For the search, we used Scopus, PubMed/Medline, and ISI Web of Science databases. The last search was carried out on October 1. The bibliographies of the studies and related reviews were included for additional references. The following search terms were used in several logical combinations for Pubmed/Medline: “("Neuroendocrine Tumors"[Mesh:NoExp] OR "Adenoma, Acidophil"[Mesh] OR "Adenoma, Basophil"[Mesh] OR "Adenoma, Chromophobe"[Mesh] OR "Apudoma"[Mesh] OR "Carcinoid Tumor"[Mesh] OR "Malignant Carcinoid Syndrome"[Mesh] OR "Carcinoma, Neu roendocrine"[Mesh] OR "Carcinoma, Medullary"[Mesh] OR "Carcinoma, Merkel Cell"[Mesh] OR "Somatostatinoma"[Mesh] OR "Vipoma"[Mesh] OR "Neurilemmoma"[Mesh] OR "Paraganglioma"[Mesh]) AND "Gastrointestinal Neoplasms"[Mesh]) OR ("Pancreatic Neoplasms"[Mesh:NoExp] AND neuroendocrine[tiab]) OR "Adenoma, Islet Cell"[Mesh] OR "Insulinoma"[Mesh] OR "Carcinoma, Islet Cell"[Mesh] OR "Gastrinoma"[Mesh] OR "Glucagonoma"[Mesh] OR ((gastroenteropancreatic OR gastro-enteric pancreatic OR gastro-entero-pancreatic OR pancreas OR pancreatic) AND (neuroendocrine AND (tumor OR tumors OR tumour OR tumours OR neoplasm OR neoplasms OR carcinoma OR carcinomas)) OR GEPNET* OR GEP-NET* OR GEPNEC* OR GEP-NEC”. The search term was also used for Scopus and the ISI-Web of Science. Thomson Reuters Endnote version X7® was used to remove the duplicate studies.

2. Study Selection

Two independent investigators (GL and CI) carried out the selection evaluating the studies in full-text form. Those articles that satisfied the eligibility criteria were evaluated in the full-text form to verify the inclusion criteria presence and the exclusion criteria absence. The inclusion criteria were based on PICOS criteria [1]: a) the "Population" was represented by the patients having non-resectable GEP-NENs; b) the "Intervention" arms were any non-surgical therapy; c) the "Control" group was the placebo arm; d) all studies reporting at least PFS and grade 3–4 toxicity;e) All phase III RCTs including at least two arms, Therefore, if two studies were reported by the same institution (or authors), either the most recent study or the one of higher quality was included. Finally, a PRISMA flowchart [2] was also formulated to demonstrate the transparency of the authors conclusions.

3. Data Collection Process and Item

The following data were extracted to describe the characteristics of each study: first author, year of publication, acronym (if present), affiliation and country, population (the type of NENs), previous treatment with SSA, chemotherapy (CHT), or other therapy, previous resection of the primary tumor, study design, the sample size of each arm and the outcomes of interest reported. As the primary endpoints, we evaluated: a) PFS as a measure of efficacy; b) the grade 3 and 4 of toxicity as a measure of safety [3]. For PFS calculation, we measured the incidence density rate (number of events for "at-risk patients" per unit time) to overcome different follow-up duration problems. This measure can be assimilated to the hazard rate for patients exposed. The rate ratio (RR) obtained from the ratio of two incidence density rates can be assimilated to the HR only for the exponential model (constant hazard functions) and absence of large differences in the average follow-up durations between the groups[4]. Dedicated software was used (GetData Graphical Digitizer®, version 2.26) to extract the crude number of events and the period of observation from Kaplan-Meier curves. The secondary efficacy-related endpoints were a) rate of objective radiological response (ORR) defined according to RECIST 1.0 or 1.1 as the sum of partial and complete response (PR+ CR) [4,5]; b) rate of progressive disease (PD) according to RECIST 1.0 or 1.1 [5,6]; c) overall survival. As secondary endpoints of safety, we evaluated: a) adverse events (AEs) and serious adverse events (SAEs) [3]; b) the "on-treatment" deaths (OTD) and the deaths drug-related (DDR) defined as all deaths for any cause and related to the drug administration, respectively; c) drug discontinuation due to AEs (DDAEs).

4. Geometry of Network

The network's geometry was plotted using one node for each arm and an edge that connected two nodes for each trial. The size of the node represents the number of patients included in each arm. The network geometry was preliminarily explored for all outcomes of interest to evaluate the presence of common nodes. When a common node was absent, the network was defined as disconnected, and this condition precludes the analysis in network modality. The network was also reported in a matrix form to obtain information about the contribution of included studies.

5. Risk of Bias within Individual Studies

The risk of bias within the individual studies was evaluated using a revised tool for assessing the risk of bias in randomized trials (Rob2) [7]. Two review authors (CR and LA.) independently assessed the risk of bias for each study using the criteria outlined in the Cochrane Handbook for Systematic Reviews of Interventions [8]. Each study was classified as follows: low risk, some concerns, or high risk.

6. Summary of Measures

All indirect and mixed estimates were reported as hazard ratios (HRs) or odds ratios (ORs) for survival and dichotomous outcomes. The HRs and ORs were expressed with 95% confidence intervals (CIs). An HR or OR with CIs crossing 1 or 0, respectively, indicated that the two competitive scenarios were equivalent. The network estimates (indirect and mixed) were reported in the forest plot [9] with CIs and predicting interval (PrI). The network results were reported first as “relative ranking probability,” which represented the probability that each arm would be the best, the second, the third, and the worst with a certain degree of uncertainty for each outcome of interest. Thus, the surface under the cumulative ranking (SUCRA) curves and mean ranks were

obtained starting these values. The SUCRA value, expressed as a percentage, showed the probability, without uncertainty, that each arm would be the best, based on the outcome analyzed [10].

7. Planned Method of Analysis, Inconsistency, Risk of Bias across the Study, and Additional Analyses

The PRISMA extension statement incorporating Network Meta-Analyses of Health Care Interventions was used to plan the analysis. Frequentist network meta-analysis was employed to compare all scenarios building a network for each outcome of interest [11]. The analysis was performed in two steps: first, all pairwise ("head-to-head") comparisons in each network were calculated to obtain the indirect and mixed estimates. Second, we calculated relative ranking probabilities, and thus SUCRA values were obtained [12]. The robustness of the networks was assessed by evaluating inconsistency, heterogeneity, and publication bias. The inconsistency was evaluated using the "loop" approach [13]. On the other hand, the restricted maximum likelihood method was used to estimate heterogeneity. The extent of heterogeneity in each network was evaluated by comparing the magnitude of a common heterogeneity variance for the network (tau [τ]) with an empirical distribution of heterogeneity variances, considering the range of expected treatment estimates (ORs and MDs). A τ value > 0.6 to was considered a high level of heterogeneity [14]. When the τ value was > 0.6, a multivariate meta-regression analysis was carried out to identify the reason for the heterogeneity in the outcome under study. Thus, all the covariates effects were reported using a small mean difference (SMD) coefficient and a *p*-value. The algorithm adopted was based on the use of maximum residual likelihood (REML). For each covariate, we described, only when significant, the following parameters: SMD coefficient with standard error (SE). The SMD coefficient ± SE was related to the change of covariate value. If SMD was different from zero value, an increased o reduction of the covariate produced a positive or negative OR modification. A two-tailed p value < 0.05 was considered statistically significant. Considering the low number of included studies in previous meta-analyses, the *p*-value was recalculated using Monte Carlo permutation [15]. The number of permutation was 500 to obtain sufficient precision [16]. Publication/reporting bias was reported using an adjusted funnel plot. Each funnel plot was tested using Begg's test to identify whether the asymmetry was attributable to the small sample size effect. A two-sided p value < 0.05 indicated a significant small sample size effect [17].

8. Results

8.1. Secondary Endpoints

The treatment with the highest probability of improving OS was Sunitinib, followed by ^{177}Lu-Dotatate plus SSA with a SUCRA value of 93.6 (mean rank = 1.4) and 87.7 (mean rank = 1.7), respectively. The worst approach was Bevacizumab plus SSA with a SUCRA value of 11.9 and a mean rank of 6.3. The ORR was evaluable only in 8 studies but in all clustered arms. The approach with the highest probability to obtain an ORR was Bevacizumab plus SSA (SUCRA = 88.3; mean rank = 1.7), followed by Sunitinib (SUCRA = 74.2; mean rank= 2.5), ^{177}Lu-Dotatate plus SSA (SUCRA = 68.6; mean rank= 2.8), IFN-α plus SSA (SUCRA = 59.0; mean rank = 3.5), the Everolimus-based one (SUCRA = 32.0; mean rank = 5.1), and SSA alone (SUCRA = 20; mean rank = 5.8). The approach with the lowest chance to obtain an ORR was a placebo (SUCRA = 6.9; mean rank = 5.8). The therapy with the highest probability to prevent a radiological progression of the disease was ^{177}Lu-Dotatate plus SSA (SUCRA = 90.6; mean rank = 1.6), followed by Bevacizumab plus SSA (SUCRA = 80.8; mean rank = 2.2),

IFN-α plus SSA (SUCRA = 61.3; mean rank = 3.3), the Everolimus-based one (SUCRA =56.9; mean rank = 3.6), Sunitinib (SUCRA = 35.0; mean rank = 4.9), and SSA alone (SUCRA = 22.8; mean rank = 5.6). The approach with the lowest chance to prevent a PD was a placebo (SUCRA = 2.6; mean rank = 6.8). The treatment with the highest probability to avoid any AE was SSA alone (SUCRA = 93.6; mean rank = 1.1), followed by placebo (SUCRA = 74.1; mean rank = 2.0), Sunitinib (SUCRA value = 3.7; mean rank = 3.7), and ^{177}Lu-Dotatate plus SSA (SUCRA = 18.3; mean rank = 4.3). Data about the IFN-α or Bevacizumab arm are lacking. When considering SAEs, the worst approach according to the model is Bevacizumab plus SSA therapy (SUCRA = 0; mean rank = 7.0) while the best is SSA alone (SUCRA = 76.4; mean rank =2.4) followed by ^{177}Lu-Dotatate plus SSA (SUCRA = 65.6; mean rank = 3.1) and placebo (SUCRA = 60.6; mean rank = 3.4). Both IFN-α (SUCRA = 19.9, mean rank = 5.8) and Everolimus arm (SUCRA = 31.0, mean rank = 5.1) have less than 50% of the chances of being the safest approach. The approach with the lowest probability of being related to OTD was Sunitinib (SUCRA = 87.3; mean rank = 1.6) followed by placebo (SUCRA = 61.3; mean rank = 2.9), SSA alone (SUCRA = 56.8, mean rank = 3.2), IFN-α plus SSA (SUCRA = 43.8; mean rank = 3.8), ^{177}Lu-Dotatate plus SSA (SUCRA = 34.9; mean rank = 4.3), and the Everolimus-based one (SUCRA = 15.9; mean rank = 5.2). OTD was not evaluable for the Bevacizumab arm. The probability of being the safest approach minimizing the DDR was over the 50% for placebo (SUCRA = 64.3; mean rank = 3.1), SSA alone (SUCRA = 58.5; mean rank = 3.5), Sunitinib (SUCRA = 58.5; mean rank = 3.5), and ^{177}Lu-Dotatate (SUCRA = 56.7; mean rank = 3.6). DDR incidence could be higher when the therapy was based on Everolimus (SUCRA = 42.0; mean rank = 4.5), IFN-α (SUCRA = 37.7; mean rank = 4.7), or Bevacizumab (SUCRA = 32.4; mean rank = 5.1). The approaches with the highest probability to avoid a "drug-discontinuation" were ^{177}Lu-Dotatate (SUCRA = 86.7; mean rank = 1.8) and placebo (SUCRA = 85.7; mean rank = 1.9) followed by SSA alone (SUCRA = 71; mean rank = 2.7), Sunitinib (SUCRA = 52.1; mean rank = 3.9), IFN-α (SUCRA = 25.0 mean rank = 5.5), Everolimus (SUCRA = 19.5; mean rank = 5.8) and Bevacizumab (SUCRA = 10.0; mean rank = 6.4).

Scheme 1. Covariates potentially source of bias and heterogeneity in included studies.

**First Author**	**Age, year (WMD, 95 %CI)**	**Male Gender (RR, 95 % CI)**	**WHO 0 PS (RR, 95 % CI)**	**G1 Neoplasm (RR, 95 % CI)**	**Lung Metastasis (RR, 95 % CI)**	**Bone Metastasis (RR, 95 % CI)**	**Extra Abdominal Metastasis (RR, 95 % CI)**	**Violation Protocol (RR, 95 % CI)**	**Primary Tumor Resection (RR, 95 % CI)**	**Previous SSA (RR, 95 % CI)**	**Previous CHT (RR, 95 % CI)**
Arnold et al.	−3.00 (−7.41 to 0.81)	1.05 (0.74 to 1.49)	NE	NE	NE	NE	NE	NE	NE	0.81 (0.29 to 2.25)	1.89 (0.61 to 5.89)
Rinke et al.	1.80 (−2.79 to 6.39)	0.89 (0.58 to 1.39)	0.94 (0.79 to 1.12)	0.34 (0.04 to 3.15)	NE	NE	NE	0.13 (0.02 to 0.98) *	1.10 (0.81 1.49)	NE	NE
Pavel et al.	0 (−1.82 to 1.82)	0.77 (0.64 to 5.93)	0.83 (0.71 to 0.97) *	0.94 (0.85 to 1.03)	1.21 (0.89 to 5.30)	1.44 (0.89 to 2.33)	NE	0.74 (0.17 to 3.27)	NR	1.03 (0.93 to 1.13)	1.19 (0.95 to 1.49)
Yao et al.	1.00 (−1.03 to 3.03)	0.92 (0.77 to 1.10)	1.02 (0.89 to 1.18)	0.97 (0.89 to 1.06)	0.97 (0.57 to 1.48)	0.42 (0.23 to 0.79) *	NE	8.83 (0.48 to 162.90)	NR	0.98 (0.81 to 1.19)	1.00 (0.82 to 1.21)
Raymond et al.	−1.00 (−3.78 to 1,78)	1.04 (0.76 to 1.42)	1.28 (0.97 to 1.68)	1.15 (0.40 to 3.29)	NE	NE	0.61 (0.39 to 0.96)*	1.98 (0.18 to 21.39)	0.98 (0.88 to 1.08)	0.93 (0.62 to 1.38)	0.92 (0.76 to 1.13)
Caplin et al.	1.10 (−1.77 to 3.97)	1.10 (0.77 to 1.30)	NE	0.98 (0.81 to 1.17)	NE	NE	NE	1.02 (0.15 to 7.10)	0.82 (0.69 to 0.97)	1.02 (0.54 to 1.93)	NE
Yao et al.	5.00 (2.56 to 7.44) *	0.79 (0.63 to 1.01)	0.97 (0.84 to 1.11)	0.94 (0.79 to 1.12)	1.06 (0.67 to 1.70)	1.32 (0.77 to 2.27)	NE	0.47 (0.03 to 7.49)	0.82 (0.69 to 0.97)*	0.96 (0.77 to 1.19)	1.11 (0.73 to 1.70)
Strosberg et al.	−1.00 (−3.47 to 1.47)	1.16 (0.89 to 1.50)	NE	0.93 (0.78 to 1.10)	2.68 (0.88 to 8.17)	1.06 (0.50 to 2.21)	NE	0.40 (0.50 to 6.63)	1.01 (0.88 to 1.15)	°°°	0.77 (0.36 to 1.61)

Yao et al.	0.10 (−2.04 to 2.24)	1.12 (0.91 to 1.38)	1.09 (0.91 to 1.32)	1.00 (0.92 to 1.09)	NE	1.12 (0.74 to 1.72)	NE	NE	NE	1.01 (0.84 to 1.19)	1.13 (0.82 to 1.57)

Legend: WMD = Weighted Mean Difference; BMI = Body Mass Index; 95 % CI= confidence interval at 95 %; RR = risk ratio; SSA = somatostatin analogues; CHT = systemic chemotherapy; NR= data Not Reported; NE = data reported but Not Extractable; * = the difference between experimental and referent arm was significant; °°°= all patients in both arms;.

Table 2. The ranking of the therapies for all outcomes.

Rank	Placebo	SSA	Everolimus	Sunitinib	^{117}Lu-DOTATATE	IFN-α	Bevacizumab
PFS							
Best	0	0	0.0	2.0	96.9	0.3	0.8
2nd	0.1	8.1	2.7	47.3	2.6	21.3	17.9
3td	0.3	19.5	11.9	14.6	0.4	31.7	21.6
4th	1.8	28.7	15.4	17.3	0.1	20.1	16.6
5th	9.8	31.1	24.0	10.0	0	12.1	13.0
6th	19.1	10.8	39.6	7.0	0	10.2	13.3
Worst	68.9	1.8	6.4	1.8	0	4.3	16.8
OS							
Best	0	0	0	59.2	40	0.7	0.1
2nd	0.3	0.1	0.9	38.2	55.8	4.0	0.7
3td	9.4	40.4	25.8	2.0	2,7	16.6	3.1
4th	20.0	27.0	31.3	0.5	1.0	9.2	11.0
5th	39.2	22.7	23.0	0.1	0.5	8.2	6.3
6th	13.5	7.8	14.3	0	0	54	10.4
Worst	17.6	2.0	4.7	0	0	7.3	68.4
ORR (CR+PR) §							
Best	0	0	0	36.3	12.5	0.1	51.1
2nd	0	0.1	0.8	19.4	26.8	21.3	31.6
3td	0.3	0.6	3.7	17.0	29.6	34.1	14.7
4th	1.6	5.3	15.2	16.7	28.5	30.9	1.8
5th	6.5	30.4	49.0	4.3	2.1	7.3	0.4
6th	22.4	40.2	29.4	3.8	0.4	3.4	0.4
Worst	69.2	23.4	1.9	2.5	0.1	2.9	0
PD							
Best	0	0	1.7	1.6	62.9	1.9	31.9
2nd	0	0.1	15.6	3.8	22.0	18.1	40.4
3td	0	0.6	20.8	9.5	11.7	42.4	15.0
4th	0.1	3.4	47.3	14.8	2.6	23.7	8.1
5th	1.1	34.7	13.6	34.9	0.7	11.7	3.3
6th	12.9	54.5	1.0	28.9	0.1	1.9	0.7
Worst	85.9	6.7	0	6.5	0	0.3	0.6
AE							
Best	8.4	89.0	0	2.6	0	*	*
2nd	78.7	9.5	0.7	7.5	3.6	*	*
3td	12.3	1.5	28.6	40.5	17.1	*	*
4th	0.6	0	48.1	25.6	25.7	*	*
5th	*	*	*	*	*	*	*
6th	*	*	*	*	*	*	*
Worst	0	0	22.6	23.8	53.6	*	*
SAE							
Best	0.4	5.5	0	85.3	8.6	0.2	0
2nd	14.6	48.2	0	9.1	27.3	0.8	0
3td	38.0	37.6	0	4.3	19.8	0.3	0
4th	45.7	8.6	1.2	1.3	42.0	1.2	0
5th	1.3	0.1	83.7	0	2.2	12.7	0
6th	0	0	15.1	0	0.1	84.8	0
Worst	0	0	0	0	0	0	100

Grade 3-4 toxicity°							
Best	27.8	7.2	0	0.1	4.4	2.3	58.2
2nd	15.6	23.3	0	1.4	7.3	45.2	7.2
3td	34.3	27.9	0	4.9	21.5	5.6	5.8
4th	15.8	31.6	0.1	18.5	18.1	6.5	9.4
5th	6.4	9.5	7.3	13.2	38.7	13.1	11.8
6th	0.1	0.5	7.1	60.4	10.0	14.8	7.1
Worst	0	0	85.5	1.5	0	12.5	0.5
On treatment deaths							
Best	6.2	6.7	0	72.7	2.3	12.1	*
2nd	43.2	18.3	0.9	13.2	7.4	17.0	*
3td	20.4	37.6	6.6	6.1	15.3	14.0	*
4th	14.9	25.2	20.6	4.1	20.2	15.0	*
5th	12.0	11.5	21.8	2.3	34.0	18.4	*
6th	*	*	*	*	*	*	*
Worst	3.3	0.7	50.1	1.6	20.8	23.5	*
Deaths Drug-related							
Best	14.9	8.4	4.2	30.4	32.4	3.6	6.1
2nd	27.6	20.5	9.1	15.7	10.0	9.4	7.7
3td	23.4	21.9	15.1	9.9	9.8	11.0	8.9
4th	15.0	23.3	19.4	8.8	8.4	14.0	11.1
5th	11.3	16.3	17.3	10.4	9.0	22.2	13.5
6th	6.6	6.5	18.1	10.6	7.7	27.2	23.3
Worst	1.2	3.1	16.8	14.2	22.7	12.6	29.4
Discontinuation for SAE							
Best	41.2	5.9	0	2.1	50.3	0.5	0
2nd	34.6	29.3	0	10.7	24.8	0.1	0.5
3td	22.8	47.7	0	13.0	15.1	1.0	0.4
4th	0.9	16.7	1.5	63.2	9.1	7.8	0.8
5th	0.5	0.2	49.1	5.0	0.7	33.1	11.4
6th	0	0.2	13.6	5.0	0	50.3	30.9
Worst	0	0	35.8	1.0	0	7.2	56.0

Legend: The probability in percentages of the therapy's ranking from best to worst is reported in the column. PFS= Progression-free survival; OS= Overall Survival; SSA = somatostatin analogs therapy; AE = Adverse Event defined according to the National Cancer Institute Common Terminology Criteria for Adverse Events; SAE = Severe Adverse Event defined according to the National Cancer Institute Common Terminology Criteria for Adverse Events; Lu = Lutetium; ORR = Objective Radiological Response; CR = Complete Response; PR = Partial Response; PD = Progressive Disease; § = evaluated according to Response Evaluation Criteria In Solid Tumors; * = not computable; ° = the datum was calculated as the number of events per patients.

Table 3. Meta-regression analysis for progression-free survival.

Legend: SMD = standard mean difference; 95 CI = confidence interval at 95%; SSA = Somatostatin analogs; IFN-α =

Covariates	Progression Free survival SMD (95 CI; *p* value)						
	**Placebo**	**SSA Alone**	**Everolimus ± SSA**	**Sunitinib Alone**	**^{177}Lu-Dotatate + SSA**	**IFN-α + SSA**	**Bevacizumab + SSA**
Low risk study (No vs Yes)	Referent	-0.33 (-1.08 to 0.41; 0.385)	*	*	*	*	*
Pancreatic NENs (No vs Yes)	Referent	0.18 (-0.63 to 1.01; 0.665)	-0.40 (-1.07 to 0.26; 0.237)	*	*	*	*
Relevant difference in mean age (No vs. Yes)	Referent	*	0.42 (-0.14 to 0.99; 0.140)	*	*	*	*
Proportion of male patients°°	Referent	-1.81 (-5.02 to 1.41; 0.271)	-0.91 (-6.78 to 4.96; 0.761)	*	*	*	*
Proportion of patients with ECOG PS equal to 0°°	Referent	*	*	*	*	*	*
Proportion of patients with G1 neoplasm°°	Referent	0-35 (-1.53 to 2.24; 0.713)	-0.68 (-36.6 to 35.2; 0.970)	*	*	*	*
At risk for difference in bone or lung metastasis rate (No vs. Yes)	Referent	-0.74 (-1.21 to -0.26; 0.002)	-0.27 (-0.61 to 0.07;0.117)	*	*	-0.61 (-122 to 121;0.992)	*
Proportion of patients with protocol violation°°	Referent	1.10 (0.24 to 5.04; 0.895)	0.99 (0.87 to 1.14;0.995)	*	*	*	*
Proportion of patients with primary tumor resection°°	Referent	0.27 (-0.57 to -1.11; 0.534)	*	*	*	*	*
Proportion of patients with previous SSA-based therapy°°	Referent	-0.26 (-1.11 to 0.58; 0.534)	*	*	*	*	*
Proportion of patients with previous Chemotherapy°°	Referent	-0.16 (-314.5 to 314; 0.999)	2.46 (-0.62 to 5.56; 0.118)	*	*	*	*
Rate of “Naïve” patients (low vs. high)	Referent	0.55 (0.33 to 0.93; 0.028)	*	*	*	*	*
Mono versus polytherapy	Referent	2.02 (-0.01 to 500;0.998)	1.13 (-0.01to 500;1.000)	*	*	*	*

Interferon- alfa; ECOG PS= Eastern Cooperative oncology group perfomance status.

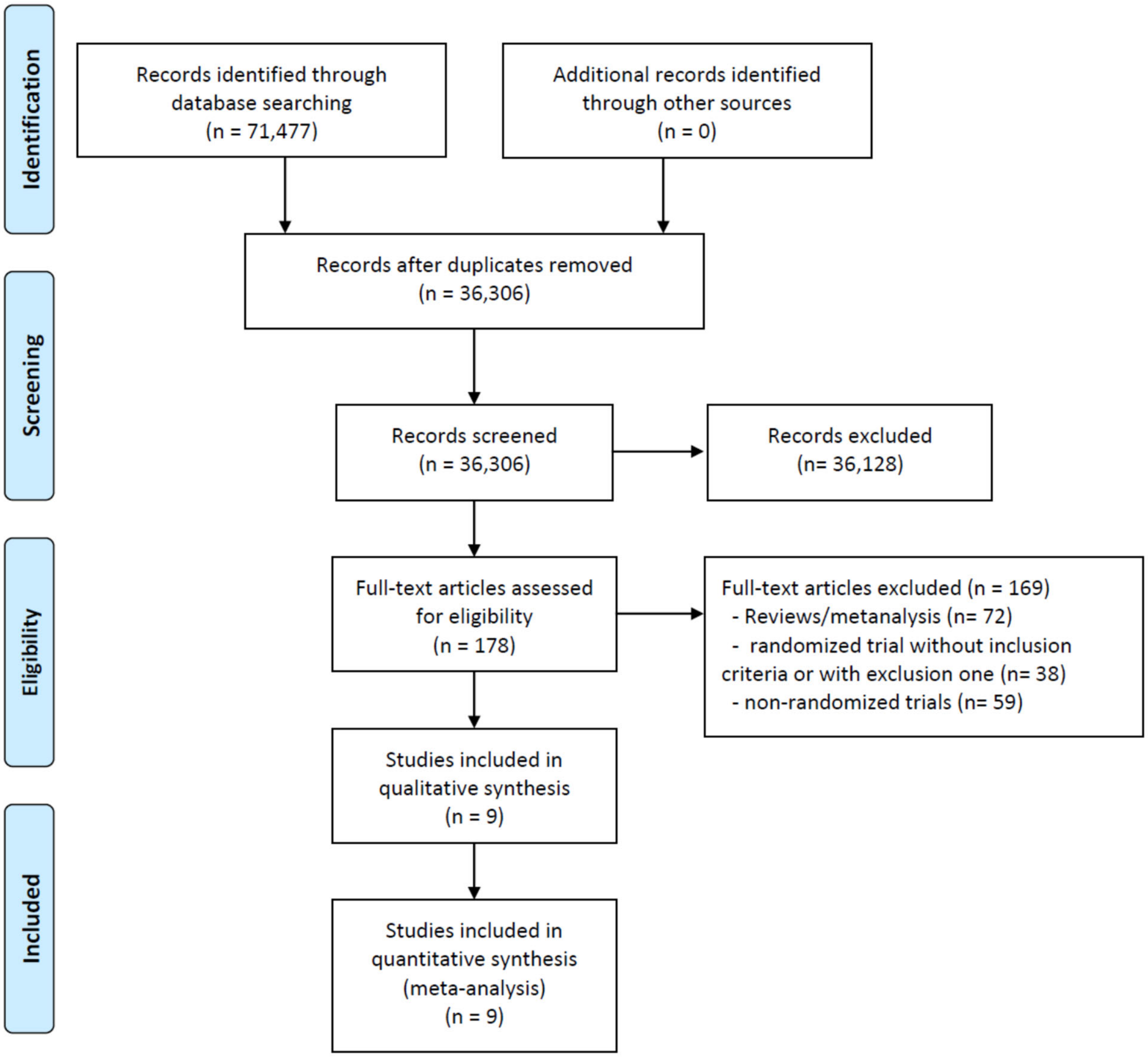

Figure 1. PRISMA Flow Diagram illustrating study selection.

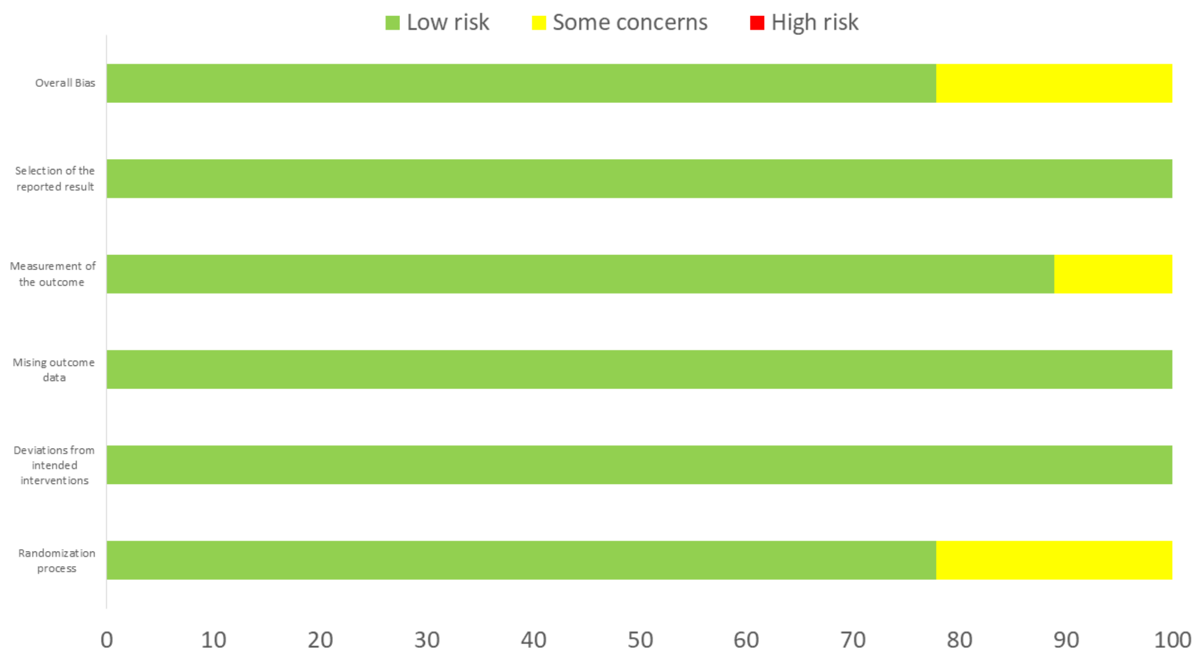

Figure 2. Quality of included study.

Figure 3 (Panel A—J): Contribution plots of all outcomes. In the columns, all available direct comparisons (comparisons evaluated in at least one study) are reported while, in the rows, the following are reported: 1) the mixed comparisons (namely the estimates already available in the literature but implemented by the network) and 2) the indirect comparisons (namely the comparisons not available in the literature but generated by the network). The table should be read from left to right; each row contains the contribution of each direct comparison in the network (mixed and indirect) estimates and, thus, the cumulative sum of the contributions is 100 (in percentages). In the plots, the contribution of each direct comparison in building the entire network is also reportedPanel B: Grade 3-4 toxicity; Panel C: Overall survival; Panel D: Objective Radiological Response; Panel E: Progressive Disease; Panel F: Adverse Event; Panel G: Severe Adverse Event; Panel H: On treatment deaths; Panel I: Deaths Drug-related; Panel J: Discontinuation for SAE

Network meta-analysis estimates	1vs2	1vs3	1vs4	2vs3	2vs5	2vs6	6vs7
	**Direct comparisons in the network**						
Mixed estimates							
1vs2	34.2	32.9		32.9			
1vs3	19.0	62.0		19.0			
1vs4			100.0				
2vs3	21.6	21.6		56.9			
2vs5					100.0		
2vs6						99.9	
6vs7							100.0
Indirect estimates							
1vs5	20.5	19.7		19.7	40.2		
1vs6	20.5	19.7		19.7		40.1	
1vs7	14.6	14.1		14.0		28.6	28.6
2vs4	20.5	19.7	40.2	19.7			
2vs7						50.0	50.0
3vs4	10.5	34.2	44.7	10.5			
3vs5	12.1	12.1		31.9	44.0		
3vs6	12.1	12.1		31.9		43.9	
3vs7	8.4	8.4		22.1		30.5	30.5
4vs5	14.6	14.1	28.6	14.1	28.6		
4vs6	14.6	14.1	28.7	14.0		28.6	
4vs7	11.3	10.9	22.3	10.9		22.3	22.3
5vs6					50.0	50.0	
5vs7					33.3	33.3	33.3
**Entire network**	11.9	13.8	12.6	15.4	12.6	21.0	12.6
**Included studies**	2	2	1	1	1	1	1

Panel A: Progression-free survival

Network meta-analysis estimates	1vs2	1vs3	1vs4	2vs3	2vs5	2vs6	6vs7
	**Direct comparisons in the network**						
Mixed estimates							
1vs2	17.3	41.4		41.4			
1vs3	12.4	75.2		12.4			
1vs4			100.0				
2vs3	13.2	13.3		73.5			
2vs5					100.0		
2vs6	0.1	0.1	0.1	0.2	0.1	99.3	[illegible]
6vs7							100.0
Indirect estimates							
1vs5	10.9	26.1		26.1	37.0		
1vs6	10.9	26.1		26.0		36.9	0.1
1vs7	7.9	19.1		19.0		26.9	27.0
2vs4	10.9	26.1	37.0	26.1			
2vs7		0.1		0.1		49.8	49.9
3vs4	6.6	40.1	46.7	6.6			
3vs5	7.1	7.1		39.4	46.5		
3vs6	7.1	7.1		39.3		46.3	0.1
3vs7	4.8	4.9		26.9		31.7	31.7
4vs5	7.9	19.0	27.0	19.0	27.0		
4vs6	7.9	19.0	27.0	19.0		26.9	0.1
4vs7	6.3	15.0	21.3	15.0		21.2	21.3
5vs6		0.1		0.1	49.9	49.8	0.1
5vs7				0.1	33.3	33.2	33.3
**Entire network**	6.7	16.4	12.4	19.2	12.4	20.5	12.4
**Included studies**	2	2	1	1	1	1	1

Panel B: Grade 3–4 toxicity

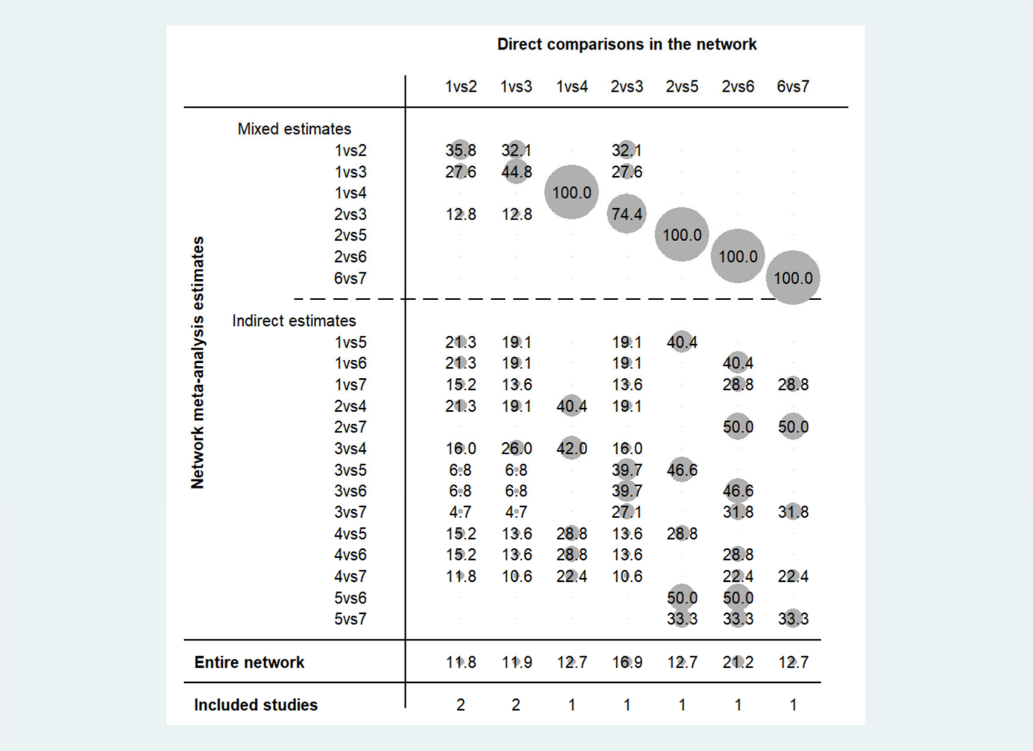

Panel C: Overall survival

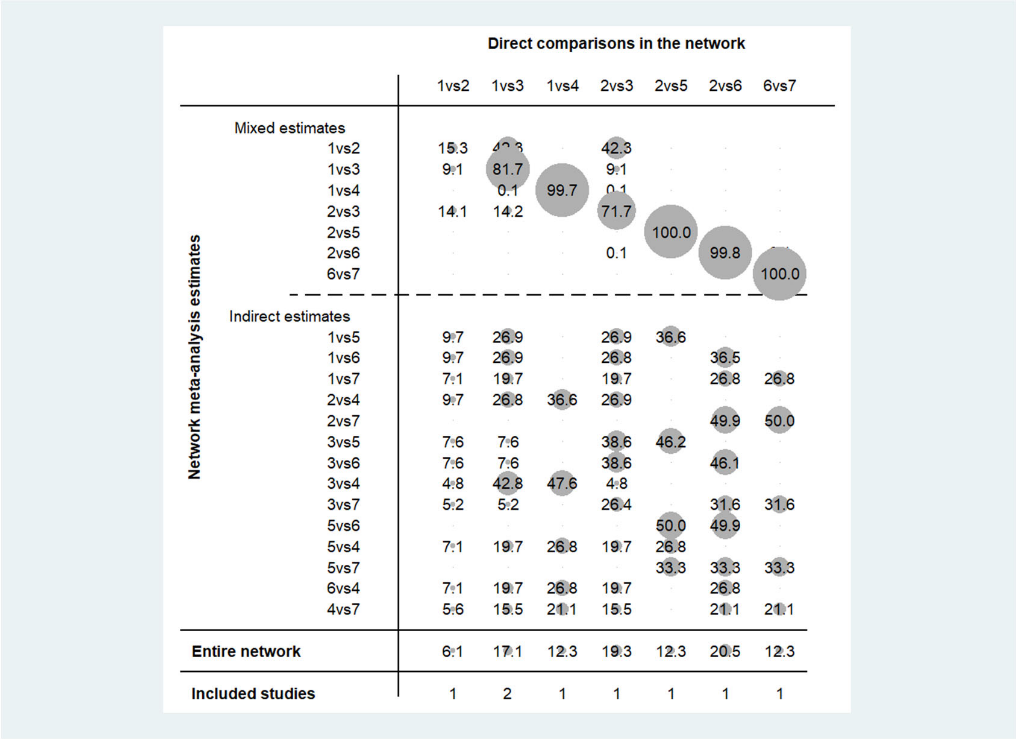

Panel D: Objective Radiological Response

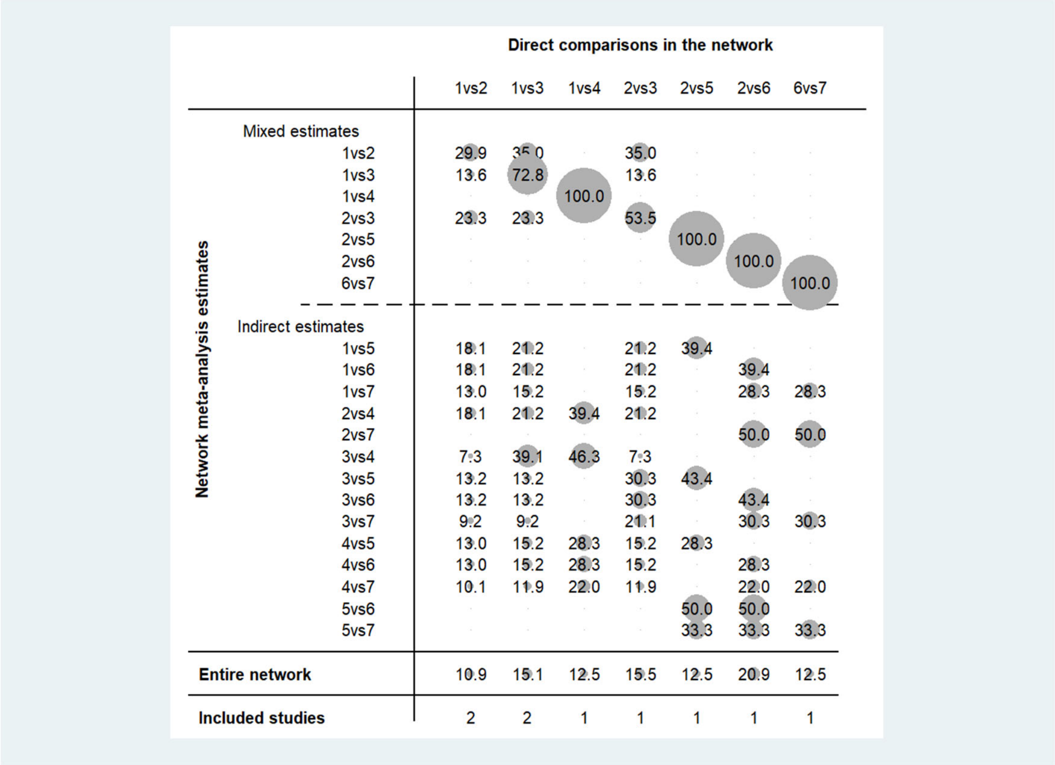

Panel E: Progressive Disease

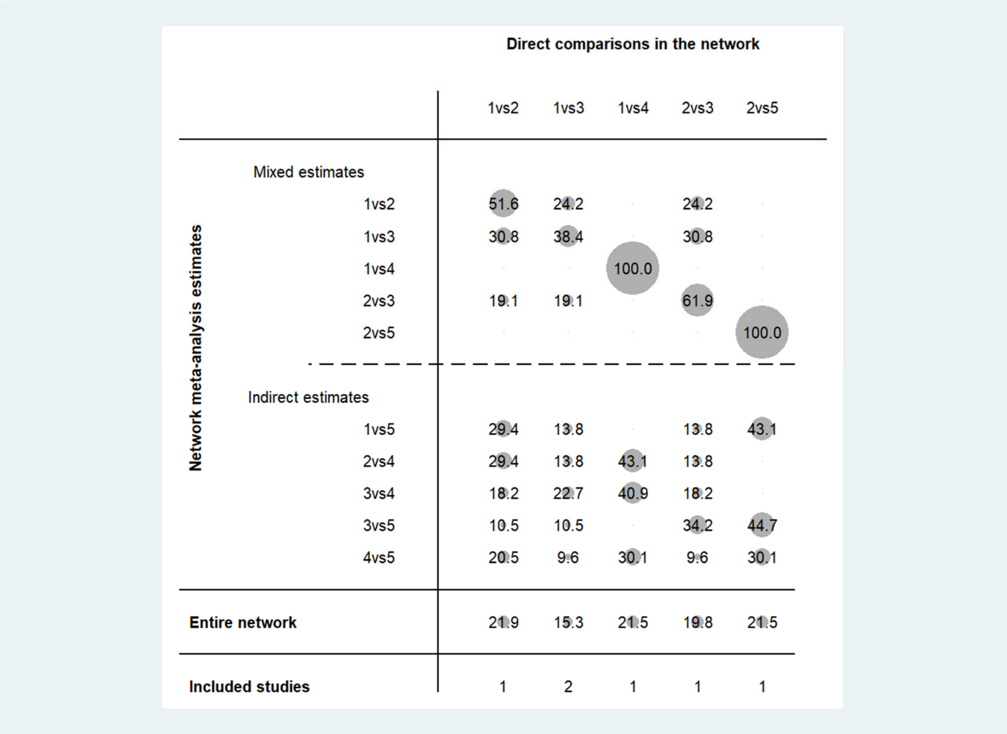

Panel F: Adverse Event

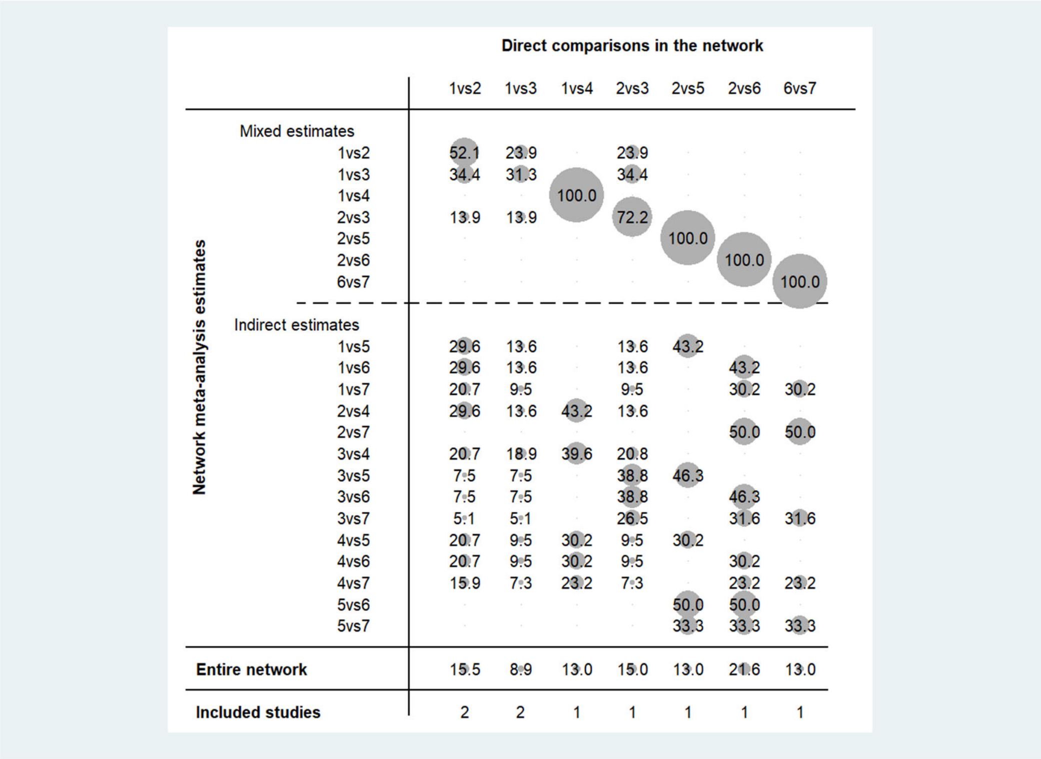

Panel G: Severe Adverse Event

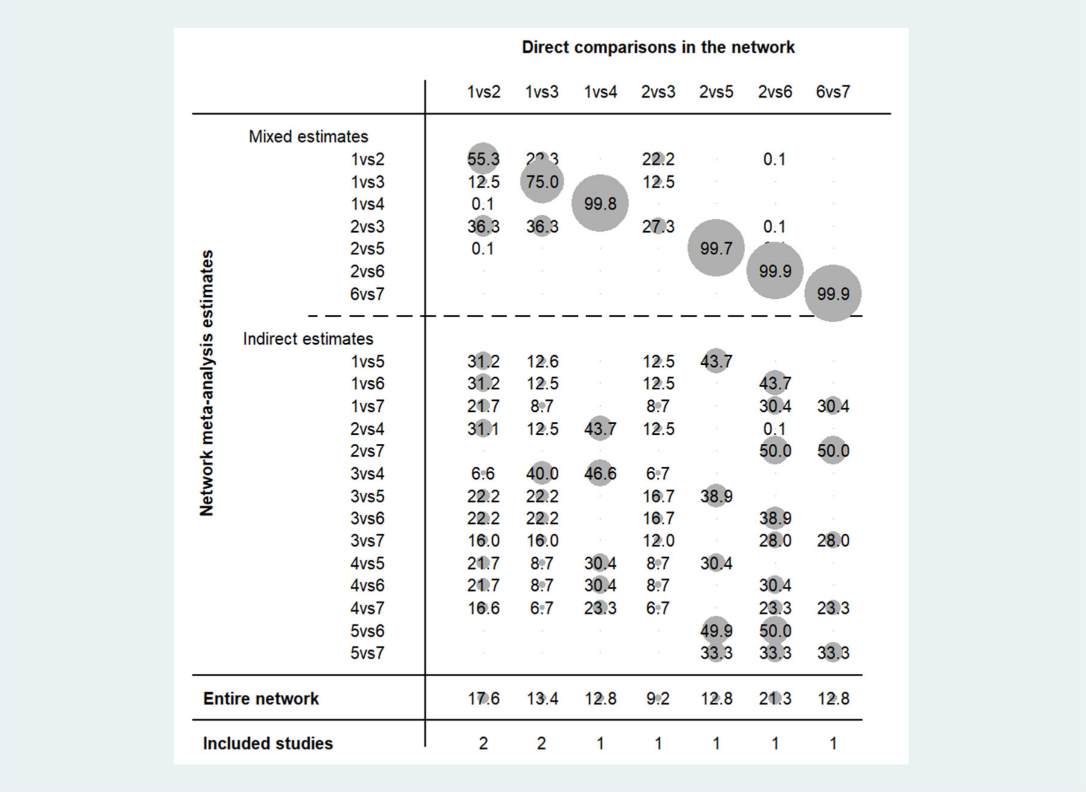

Panel H: On treatment deaths.

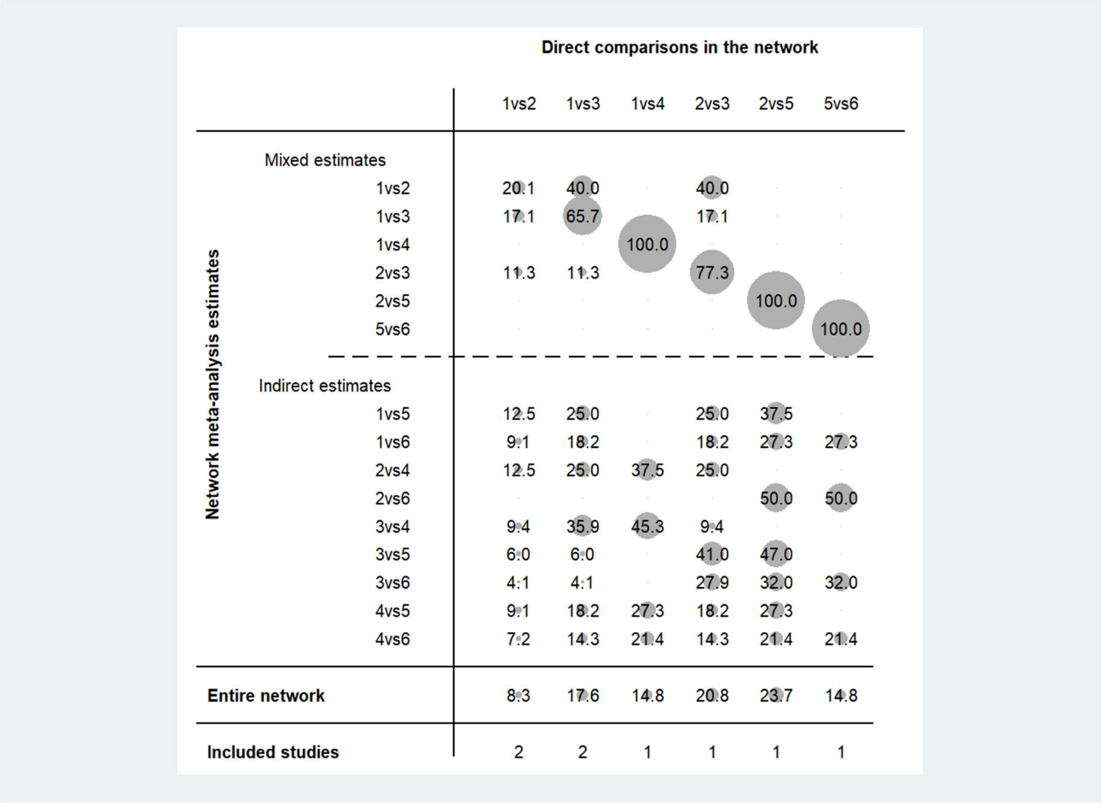

Panel I: Deaths Drug-related.

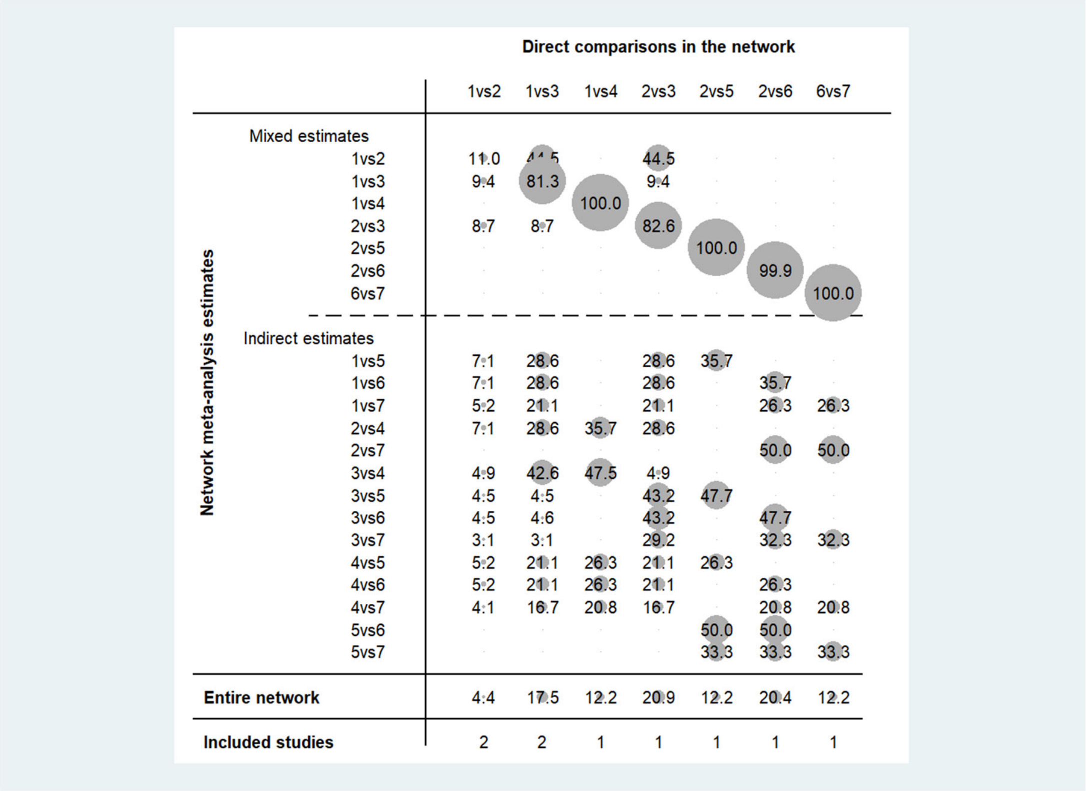

Panel J: Discontinuation for SAE.

Figure 4 (Panel A—J): Forest plots of all outcomes. The results are reported as Odds ratios (ORs) and 95% confidence intervals (CIs). The blue line (line of null effect) is equal to 1. The solid black lines represent the CIs while the diamond summarises the ORs. For each pairwise comparison, the forest plot should be read as following: if the diamond with the entire CIs did not reach the blue line of null effect, there is a significant difference. If the entire CI is on the left of the null effect, the mortality rate is significantly higher in the "intervention arm" while, when the entire CI is on the right, the event is statistically more frequent in the "reference arm." When the entire CI crosses the null effect line, the difference between the two procedures compared is not statistically significant. Besides, a red line reports the Predictive Interval (PrI), namely the interval within which the estimate of a future study is expected to be. Panel A: Progression-free survival; Panel B: Grade 3-4 toxicity; Panel C: Overall survival; Panel D: Objective Radiological Response; Panel E: Progressive Disease; Panel F: Adverse Event; Panel G: Severe Adverse Event; Panel H: On treatment deaths; Panel I: Deaths Drug-related; Panel J: Discontinuation for Severe Adverse Event.

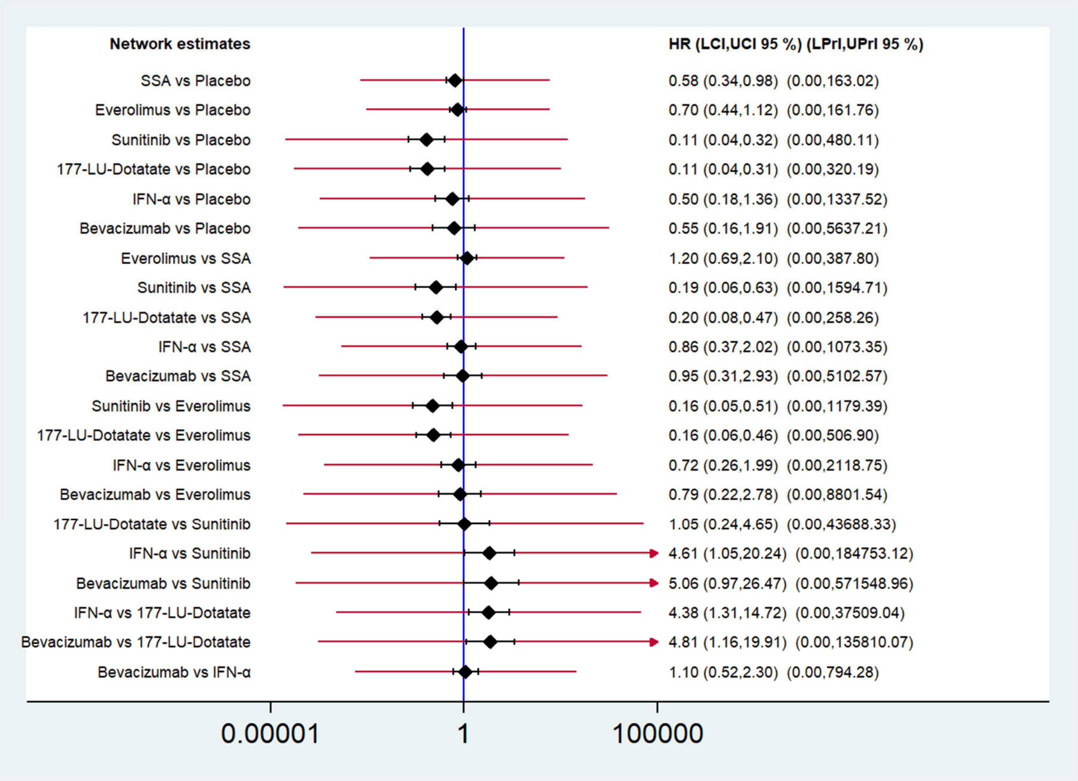

Panel A: Progression-free survival

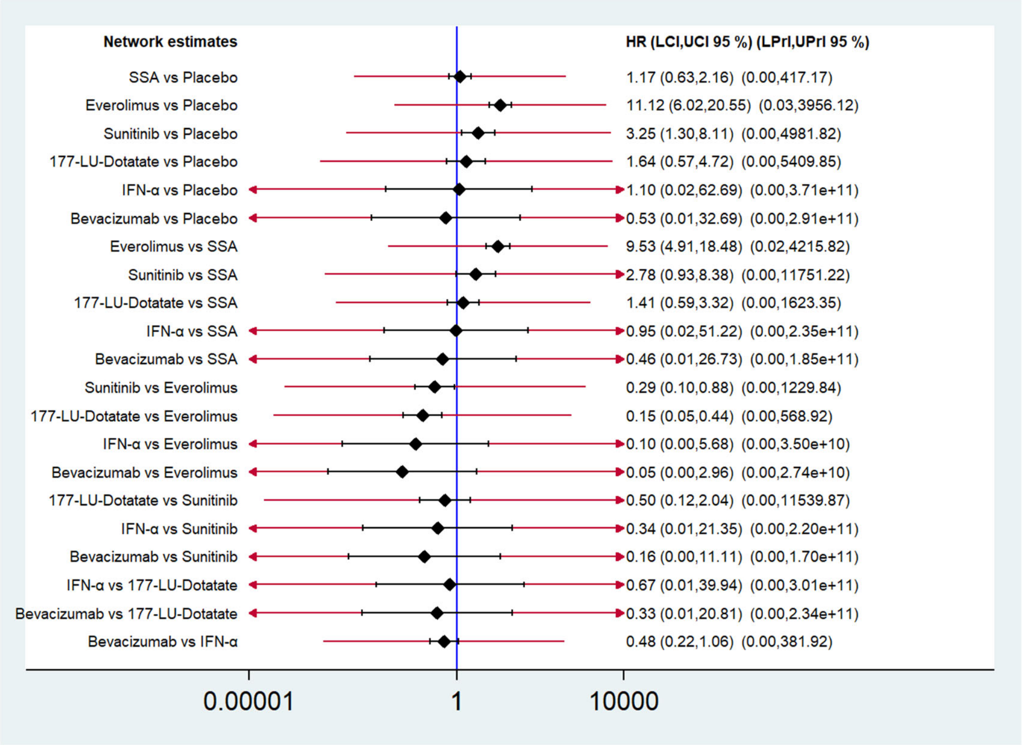

Panel B: Grade 3–4 toxicity

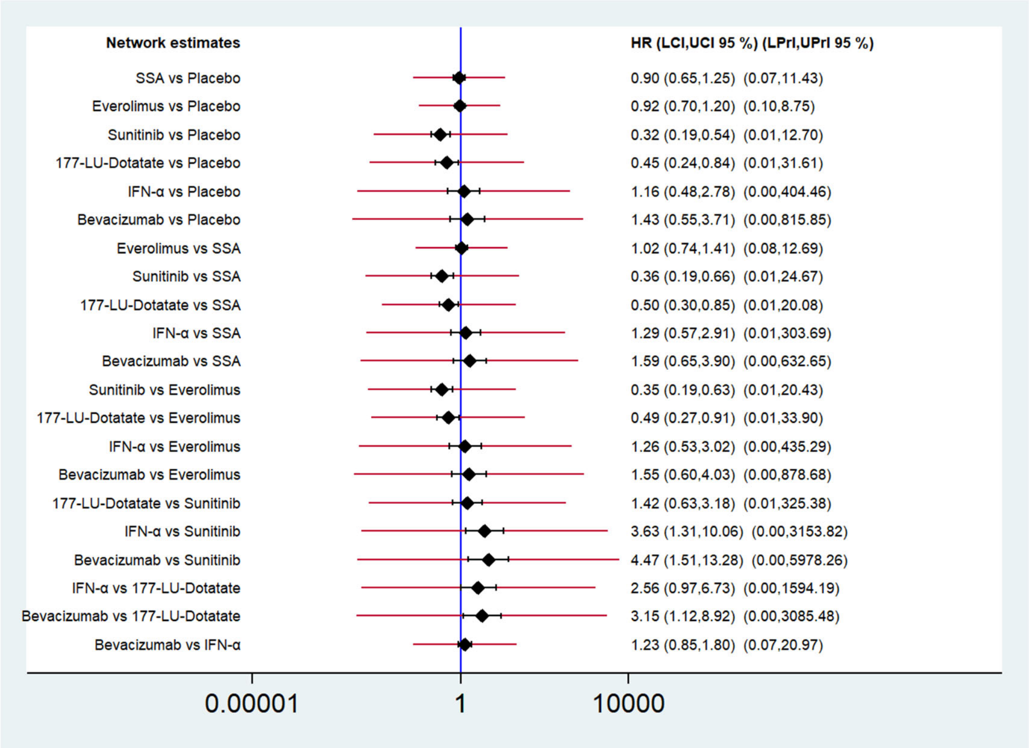

Panel C: Overall survival

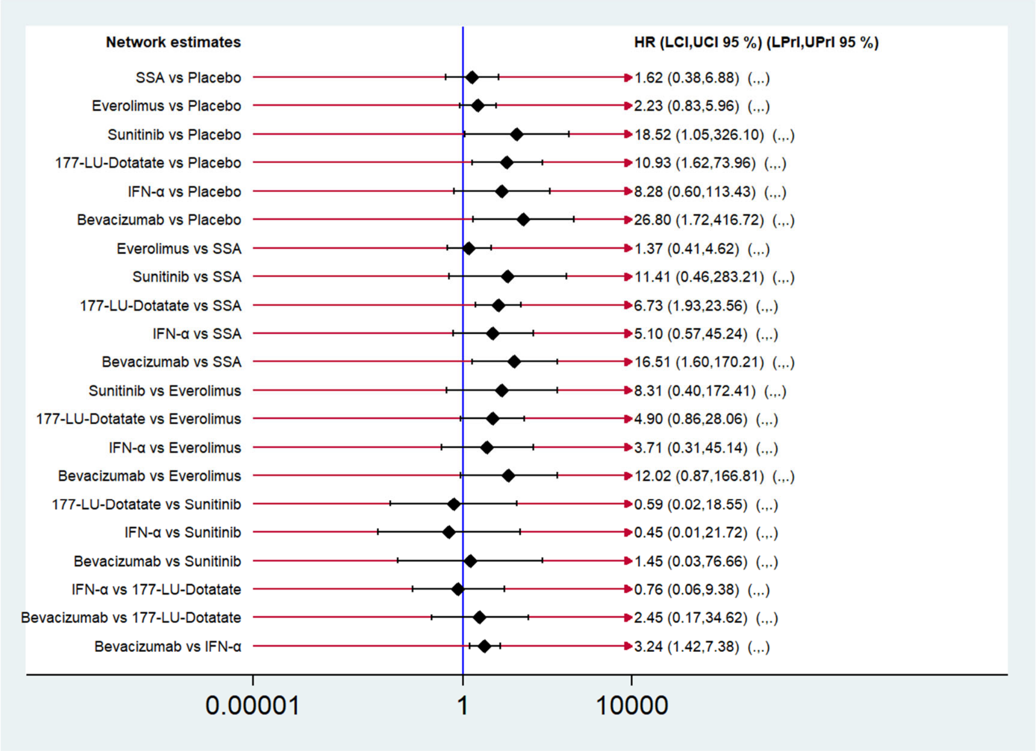

Panel D: Objective Radiological Response

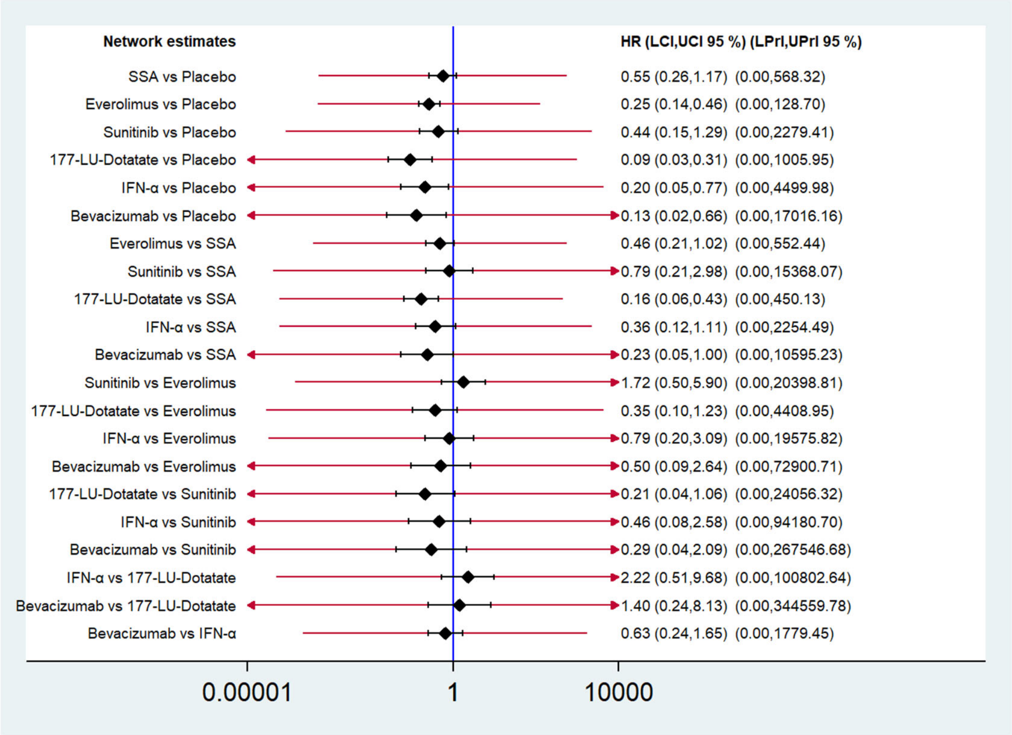

Panel E: Progressive Disease

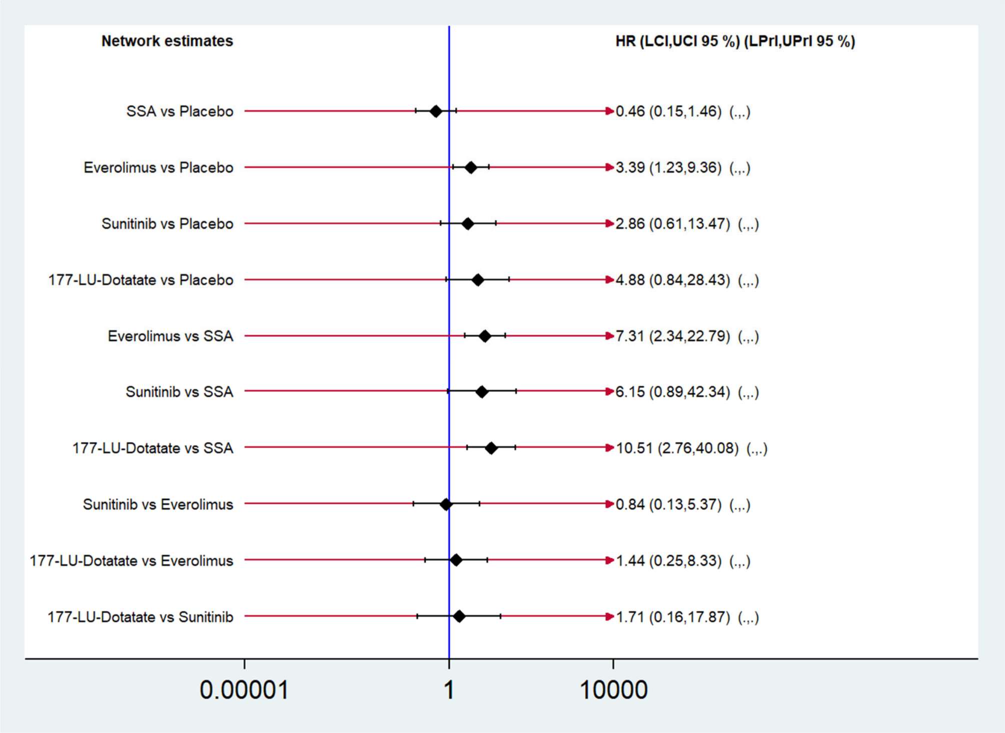

Panel F: Adverse Event

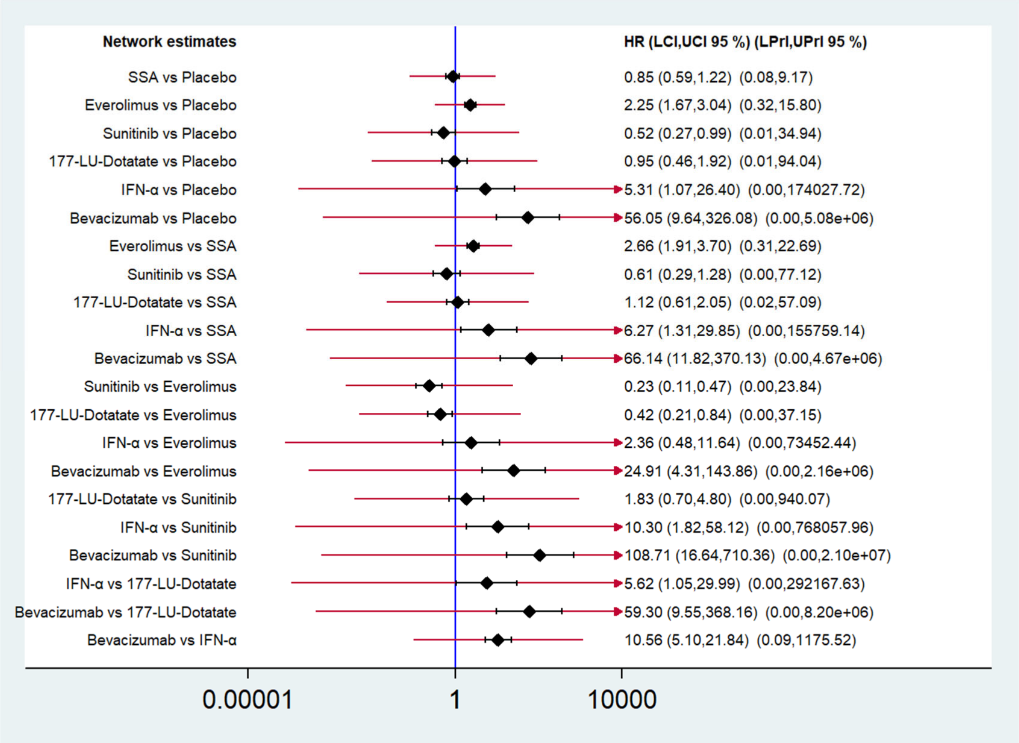

Panel G: Severe Adverse Event

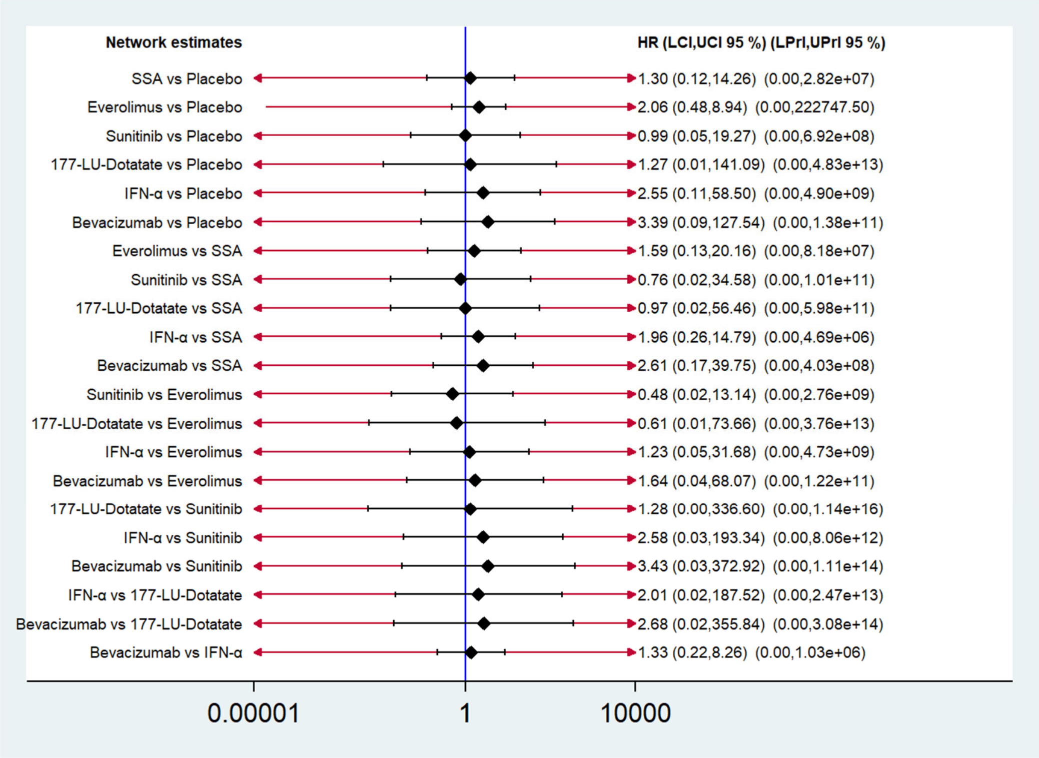

Panel I: Deaths Drug-related

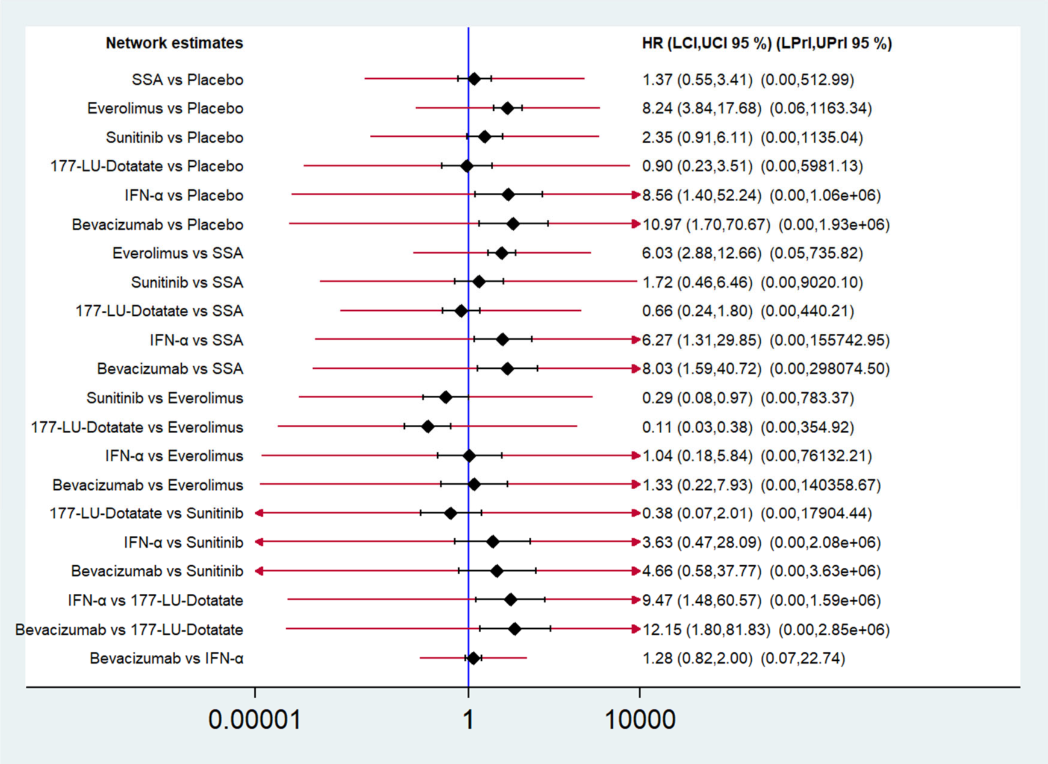

Panel J: Discontinuation for Severe Adverse Event

Figure 5: Funnel plots of the network estimates of all outcomes. In the comparison-adjusted funnel plot, the horizontal axis shows the difference of each i-study estimate

YiXY from the summary effect for the respective comparison (YiXY-µXY). In contrast, the vertical axis presents a measure of the dispersion of YiXY, namely the standard error of the effect size. The red line shows the null hypothesis. Each point represents a direct comparison: different colors correspond to different comparisons. The dashed black line represents the 95% confidence interval. The horizontal line represents the regression line; the sky blue regression line demonstrates no asymmetry; Arm 1= Placebo; Arm 2= octreotide analogs (SSA) alone; Arm 3 = Everolimus ± SSA; Arm 4 = Sunitinib; Arm 5 = ^{177}Lu-Dotatate plus SSA; Arm 6 = Interferon alfa plus SSA; Arm 7= Bevacizumab plus SSA.

Panel A: Progression-free survival; Panel B: Grade 3-4 toxicity; Panel C: Overall survival; Panel D: Objective Radiological Response; Panel E: Progressive Disease; Panel F: Adverse Event; Panel G: Severe Adverse Event; Panel H: On treatment deaths; Panel I: Deaths Drug-related; Panel J: Discontinuation for Severe Adverse Event

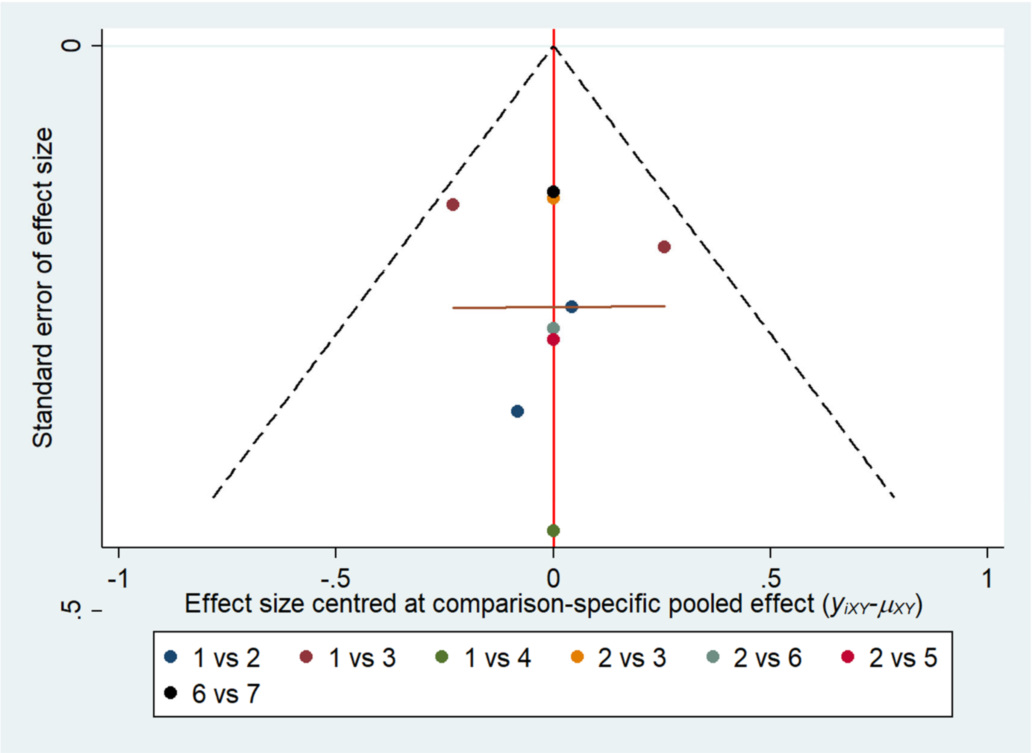

Panel A: Progression-free survival

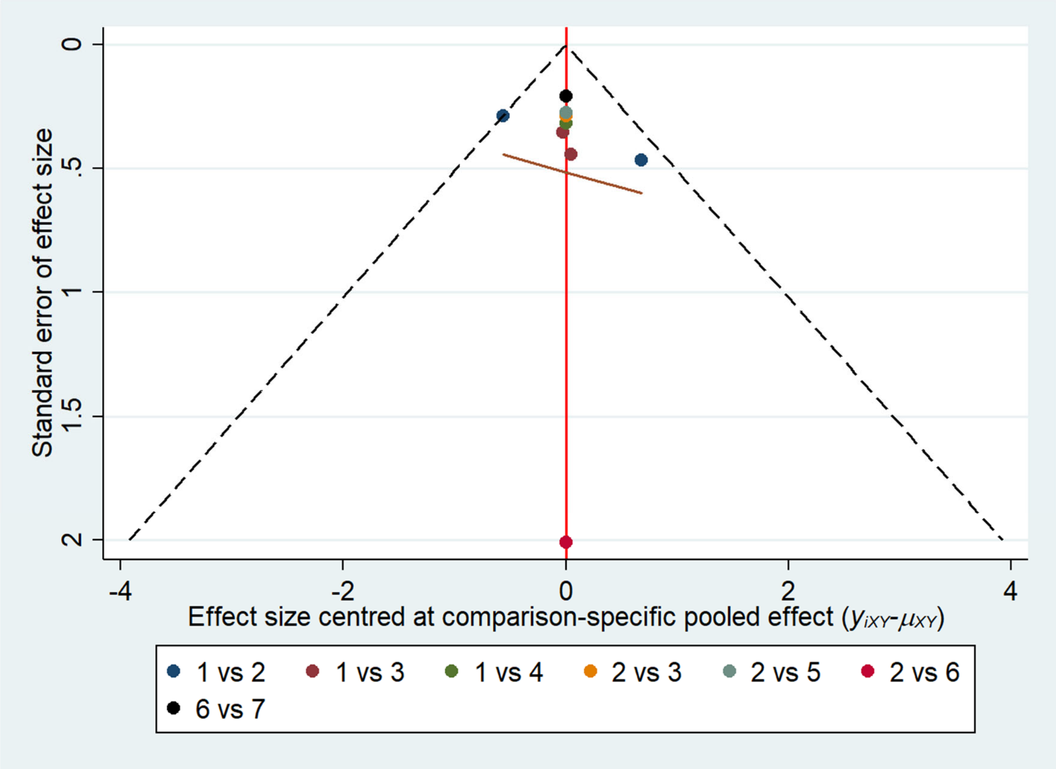

Panel B: Grade 3–4 toxicity

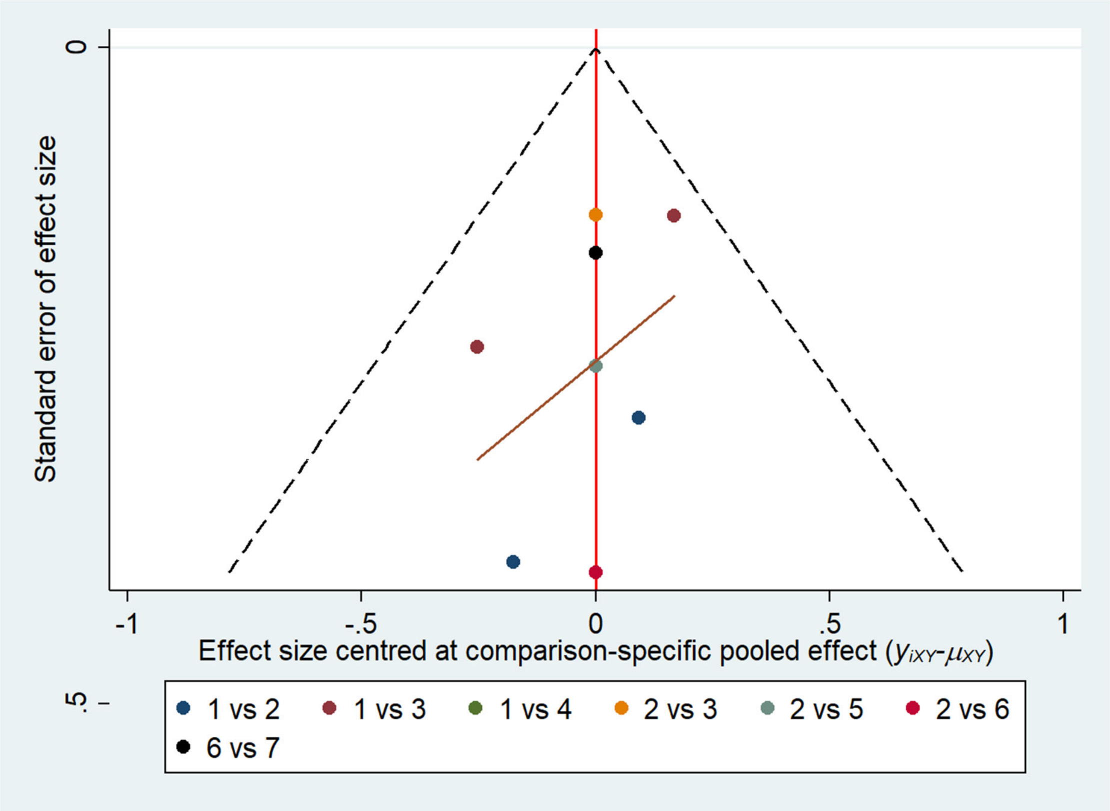

Panel C: Overall survival

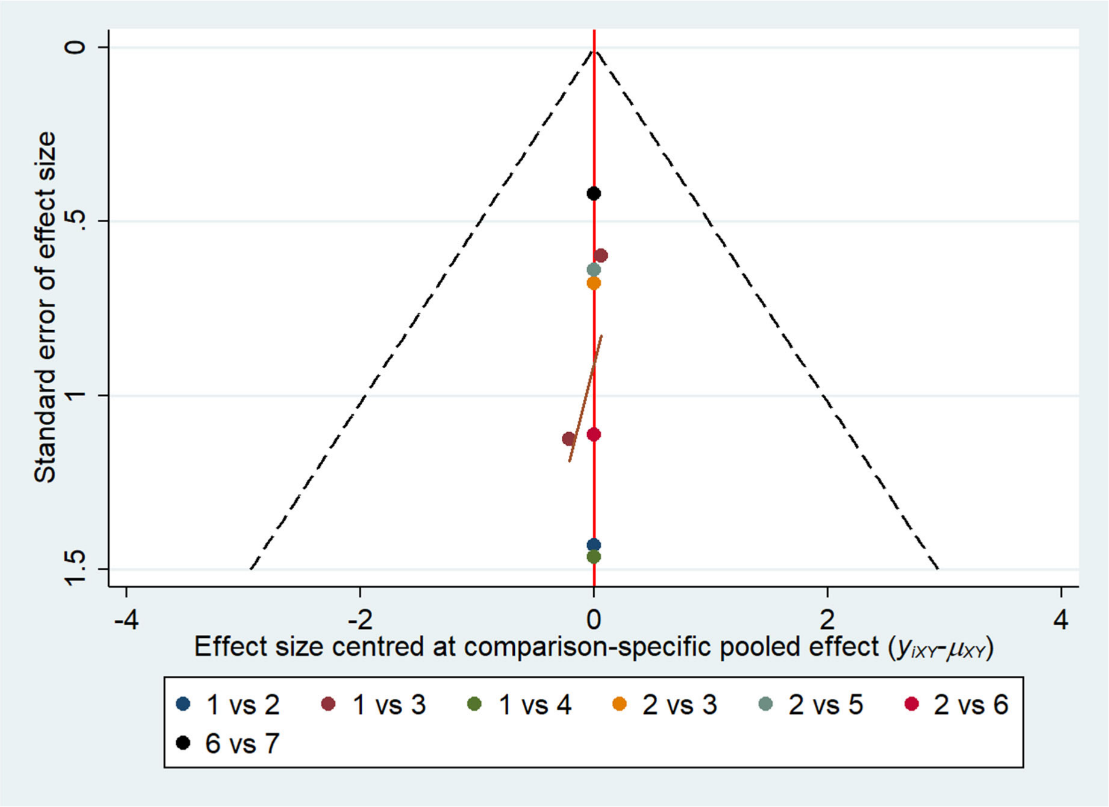

Panel D: Objective Radiological Response

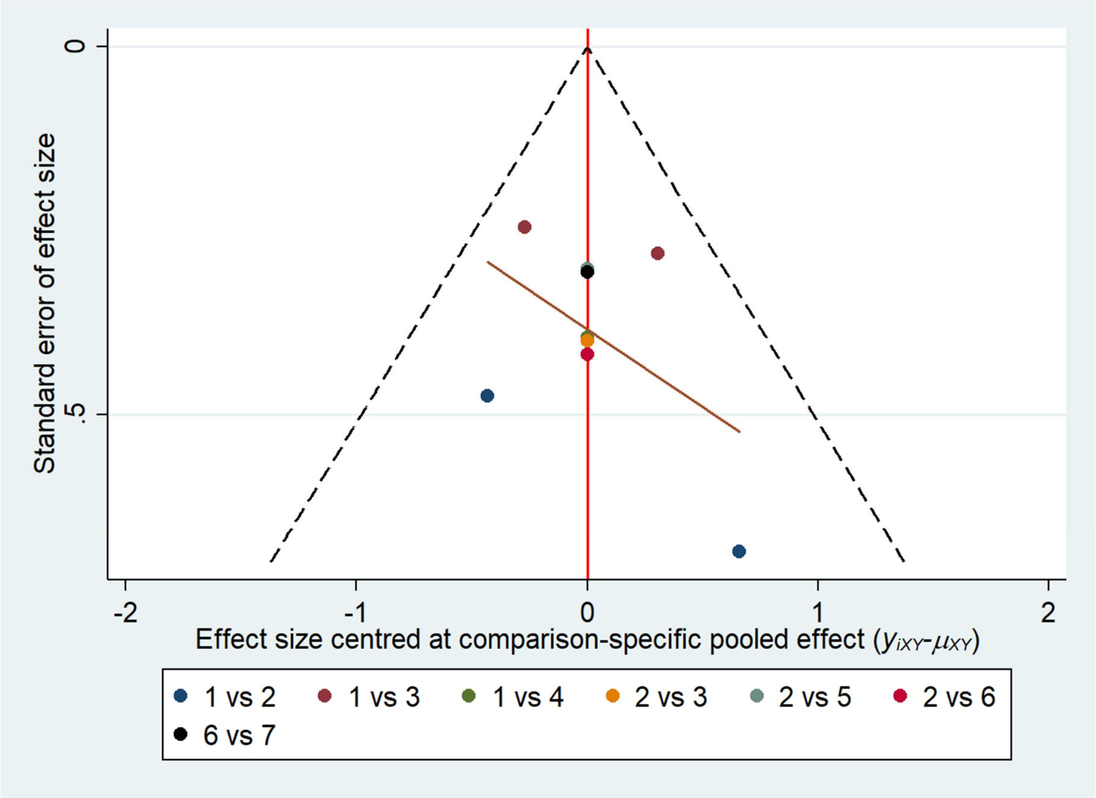

Panel E: Progressive Disease

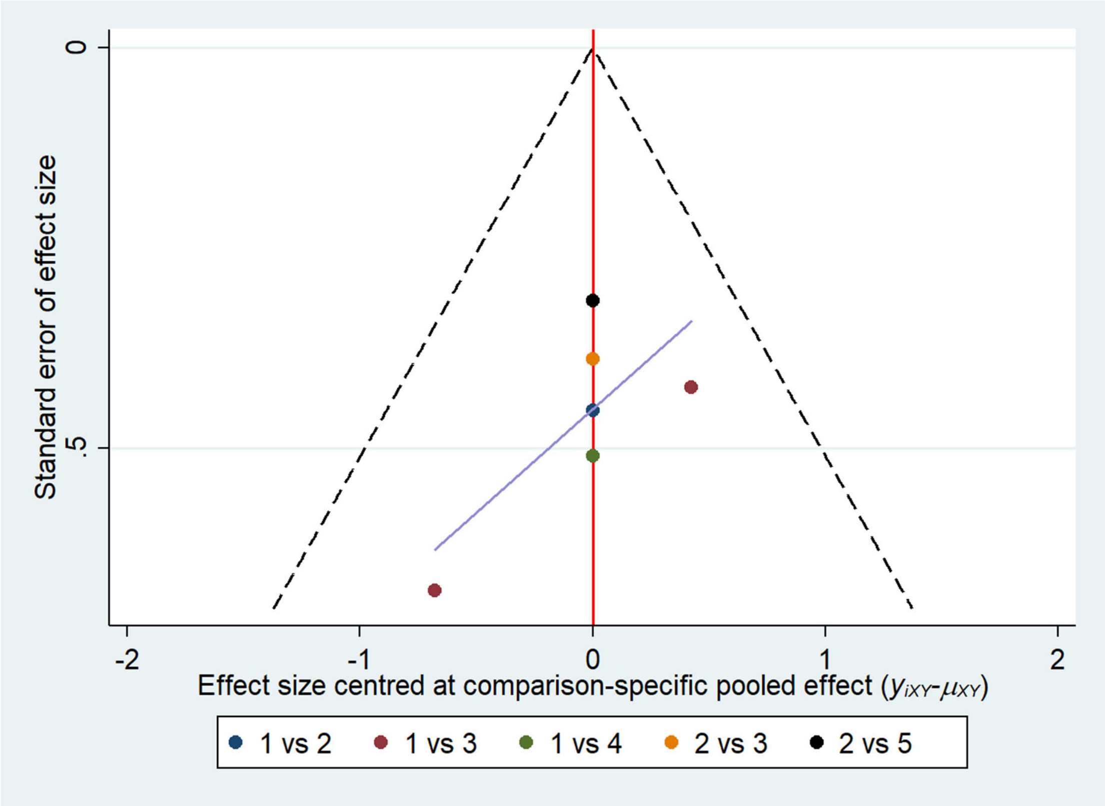

Panel F: Adverse Event

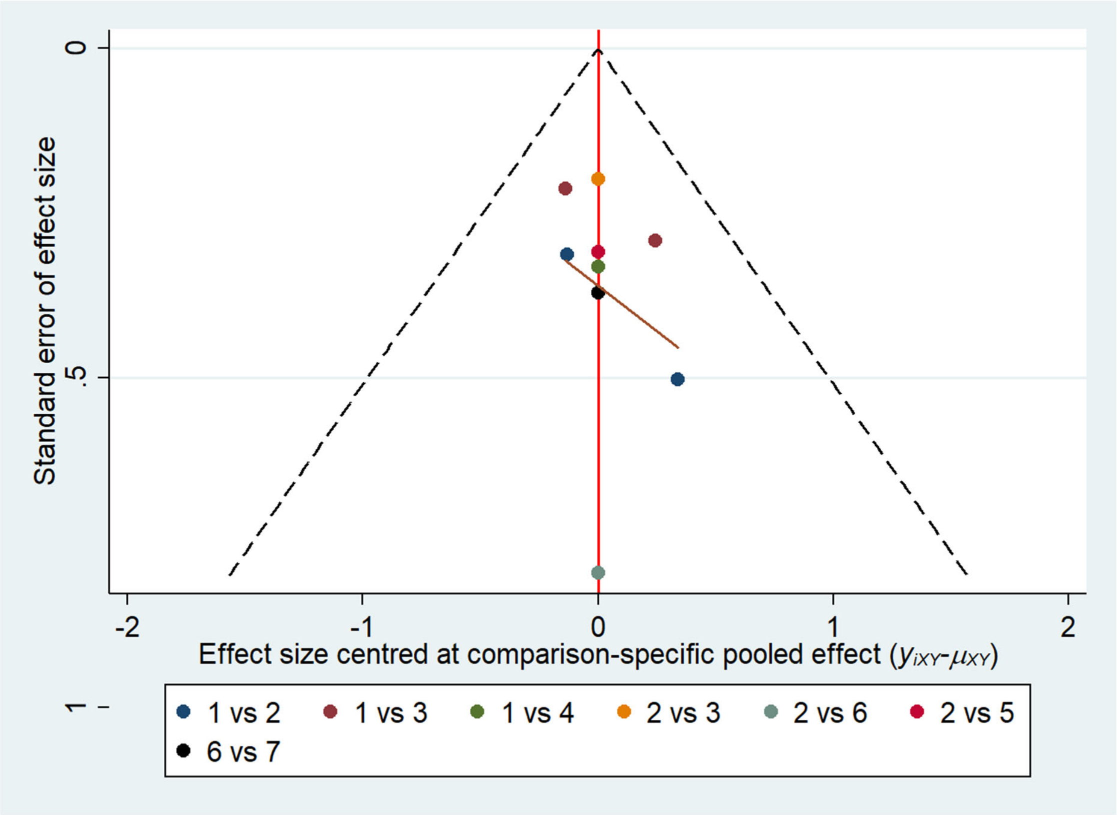

Panel G: Severe Adverse Event

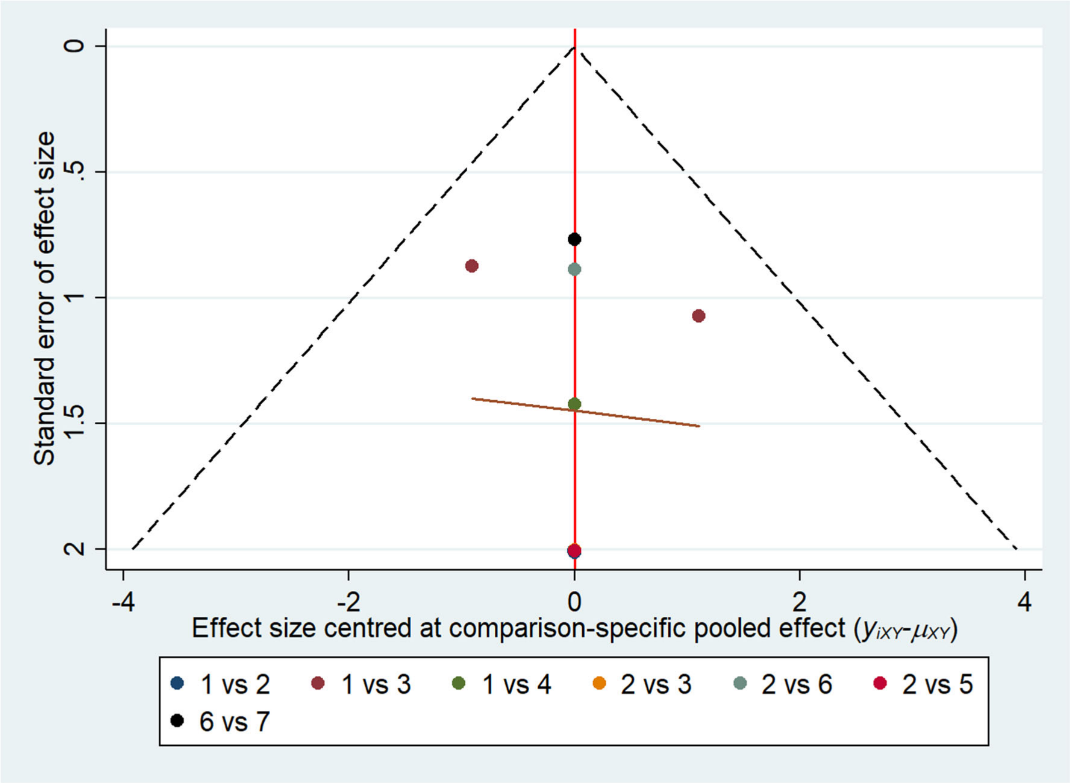

Panel I: Deaths Drug-related

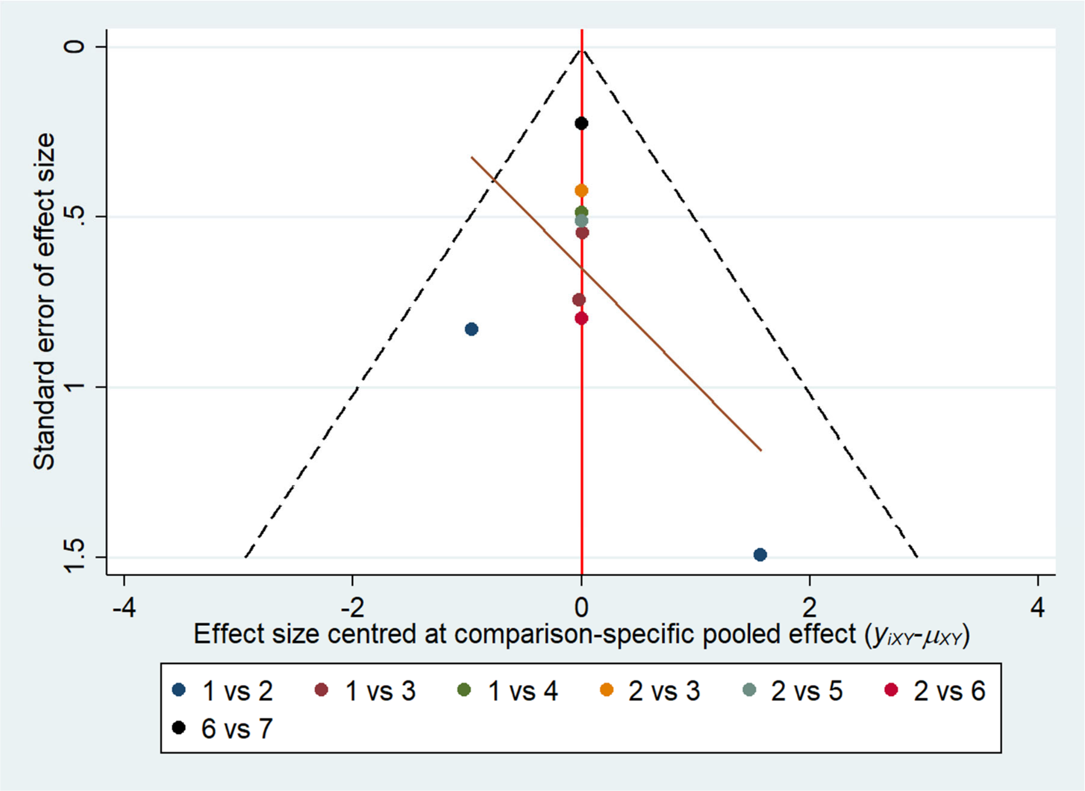

Panel J: Discontinuation for Severe Adverse Event

References

1. Eriksen, M.B.; Frandsen, T.F. The impact of patient, intervention, comparison, outcome (PICO) as a search strategy tool on literature search quality: a systematic review. *J. Med Libr. Assoc.* **2018**, *106*, 420–431, doi:10.5195/jmla.2018.345.
2. Liberati, A.; Altman, D.G.; Tetzlaff, J.; Mulrow, C.D.; Gøtzsche, P.C.; Ioannidis, J.P.; Clarke, M.F.; Devereaux, P.J.; Kleijnen, J.; Moher, D. The PRISMA Statement for Reporting Systematic Reviews and Meta-Analyses of Studies That Evaluate Health Care Interventions: Explanation and Elaboration. *Ann. Intern. Med.* **2009**, *151*, W-65, doi:10.7326/0003-4819-151-4-200908180-00136.
3 - https://ctep.cancer.gov/protocolDevelopment/electronic_applications/docs/aeguidelines.pdf.
4. Bender, R.; Beckmann, L. Limitations of the incidence density ratio as approximation of the hazard ratio. *Trials* **2019**, *20*, 1–8, doi:10.1186/s13063-019-3590-2.
5. Therasse, P.; Arbuck, S.G.; Eisenhauer, E.A.; Wanders, J.; Kaplan, R.S.; Rubinstein, L.; Verweij, J.; Van Glabbeke, M.; Van Oosterom, A.T.; Christian, M.C.; et al. New Guidelines to Evaluate the Response to Treatment in Solid Tumors. *J. Natl. Cancer Inst.* **2000**, *92*, 205–216, doi:10.1093/jnci/92.3.205.
6. Eisenhauer, E.A.; Therasse, P.; Bogaerts, J.; Schwartz, L.H.; Sargent, D.; Ford, R.; Dancey, J.; Arbuck, S.; Gwyther, S.; Mooney, M.; et al. New response evaluation criteria in solid tumours: Revised RECIST guideline (version 1.1). *Eur. J. Cancer* **2009**, *45*, 228–247, doi:10.1016/j.ejca.2008.10.026.
7. Sterne, J.A.C.; Savović, J.; Page, M.J.; Elbers, R.G.; Blencowe, N.S.; Boutron, I.; Cates, C.J.; Cheng, H.-Y.; Corbett, M.S.; Eldridge, S.M.; et al. RoB 2: a revised tool for assessing risk of bias in randomised trials. *BMJ* **2019**, *366*, l4898, doi:10.1136/bmj.l4898.
8. Higgins JPT, Green, S. Cochrane Handbook for Systematic Reviews of Interventions Version 5.1.0 [updated March 2011]. The Cochrane Collaboration, 2011. Available from http://handbook.cochrane.org. Accessed January 3, 2019.
9. Chaimani, A.; Higgins, J.P.T.; Mavridis, D.; Spyridonos, P.; Salanti, G. Graphical Tools for Network Meta-Analysis in STATA. *PLOS ONE* **2013**, *8*, e76654, doi:10.1371/journal.pone.0076654.
10. Bucher, H.C.; Guyatt, G.H.; Griffith, L.E.; Walter, S.D. The results of direct and indirect treatment comparisons in meta-analysis of randomized controlled trials. *J. Clin. Epidemiology* **1997**, *50*, 683–691, doi:10.1016/s0895-4356(97)00049-8.
11. Salanti, G.; Ades, A.; Ioannidis, J.P. Graphical methods and numerical summaries for presenting results from multiple-treatment meta-analysis: an overview and tutorial. *J. Clin. Epidemiology* **2011**, *64*, 163–171, doi:10.1016/j.jclinepi.2010.03.016.
12. Mills, E.J.; Ioannidis, J.P.A.; Thorlund, K.; Schünemann, H.J.; Puhan, M.A.; Guyatt, G.H. How to Use an Article Reporting a Multiple Treatment Comparison Meta-analysis. *JAMA* **2012**, *308*, 1246–1253, doi:10.1001/2012.jama.11228.
13. Shim, S.; Yoon, B.-H.; Shin, I.-S.; Bae, J.-M. Network meta-analysis: application and practice using Stata. *Epidemiology Heal.* **2017**, *39*, e2017047, doi:10.4178/epih.e2017047.
14. Turner, R.M.; Davey, J.; Clarke, M.J.; Thompson, S.G.; Higgins, J.P. Predicting the extent of heterogeneity in meta-analysis, using empirical data from the Cochrane Database of Systematic Reviews. *Int. J. Epidemiology* **2012**, *41*, 818–827, doi:10.1093/ije/dys041.
15. Higgins, J.P.T.; Thompson, S.G. Controlling the risk of spurious findings from meta-regression. *Stat. Med.* **2004**, *23*, 1663–1682, doi:10.1002/sim.1752.
16. Manly BFJ. 2006. Randomization, Bootstrap and Monte Carlo Methods in Biology. 3rd ed. Boca Raton, FL: Chapman & Hall/CRC.
17. Egger, M.; Smith, G.D.; Schneider, M.; Minder, C. Bias in meta-analysis detected by a simple, graphical test. *BMJ* **1997**, *315*, 629–634, doi:10.1136/bmj.315.7109.629.
